# A Detailed, Hierarchical Study of *Giardia lamblia*'s Ventral Disc Reveals Novel Microtubule-Associated Protein Complexes

**DOI:** 10.1371/journal.pone.0043783

**Published:** 2012-09-11

**Authors:** Cindi L. Schwartz, John M. Heumann, Scott C. Dawson, Andreas Hoenger

**Affiliations:** 1 Boulder Lab for 3-D Electron Microscopy of Cells, Department of MCD Biology, University of Colorado, Boulder, Colorado, United States of America; 2 Department Microbiology, University of California Davis, Davis, California, United States of America; University of Oklahoma Health Sciences Center, United States of America

## Abstract

*Giardia lamblia* is a flagellated, unicellular parasite of mammals infecting over one billion people worldwide. *Giardia's* two-stage life cycle includes a motile trophozoite stage that colonizes the host small intestine and an infectious cyst form that can persist in the environment. Similar to many eukaryotic cells, *Giardia* contains several complex microtubule arrays that are involved in motility, chromosome segregation, organelle transport, maintenance of cell shape and transformation between the two life cycle stages. *Giardia* trophozoites also possess a unique spiral microtubule array, the ventral disc, made of approximately 50 parallel microtubules and associated microribbons, as well as a variety of associated proteins. The ventral disc maintains trophozoite attachment to the host intestinal epithelium. With the help of a combined SEM/microtome based slice and view method called 3View® (Gatan Inc., Pleasanton, CA), we present an entire trophozoite cell reconstruction and describe the arrangement of the major cytoskeletal elements. To aid in future analyses of disc-mediated attachment, we used electron-tomography of freeze-substituted, plastic-embedded trophozoites to explore the detailed architecture of ventral disc microtubules and their associated components. Lastly, we examined the disc microtubule array in three dimensions in unprecedented detail using cryo-electron tomography combined with internal sub-tomogram volume averaging of repetitive domains. We discovered details of protein complexes stabilizing microtubules by attachment to their inner and outer wall. A unique tri-laminar microribbon structure is attached vertically to the disc microtubules and is connected to neighboring microribbons via crossbridges. This work provides novel insight into the structure of the ventral disc microtubules, microribbons and associated proteins. Knowledge of the components comprising these structures and their three-dimensional organization is crucial toward understanding how attachment via the ventral disc occurs *in vivo*.

## Introduction

Giardiasis is the most common cause of protozoan intestinal infection worldwide [1], and has been included in the World Health Organization (WHO) Neglected Diseases Initiative as one of a group of diseases of global importance that are linked with poverty and limit development and socio-economic improvements [1], [2]. *Giardia* has a two-stage life cycle, and acute giardiasis results from the ingestion of the cyst form and subsequent colonization and attachment of the flagellated trophozoite form to the small intestine via the ventral disc [3]. The emergence of drug resistance in *Giardia* isolates [4] highlights the need for further research to define the mechanisms of giardial virulence and identify novel anti-giardial compounds.

Beyond canonical axonemes and spindle assemblies, many parasitic protists have evolved elaborate microtubule-based arrays that enable specialized functions throughout their complex life cycles. Each of these arrays have a unique supramolecular architecture, including the subpellicular microtubule array of apicomplexans [5] (e.g., the conoid of *Toxoplasma* [6]) and trypanosomes [7]. These elaborate structures are composed of numerous and novel microtubule-associated proteins (MAPs) that presumably enable the unique structural and dynamic functions of these microtubule-based arrays. Very few of these specialized microtubule assemblies have been imaged in three dimensions *in situ*, but understanding their organization in detail is critical toward ascertaining the function of these organelles in mediating the pathogenesis of the parasites.

**Figure 1:**
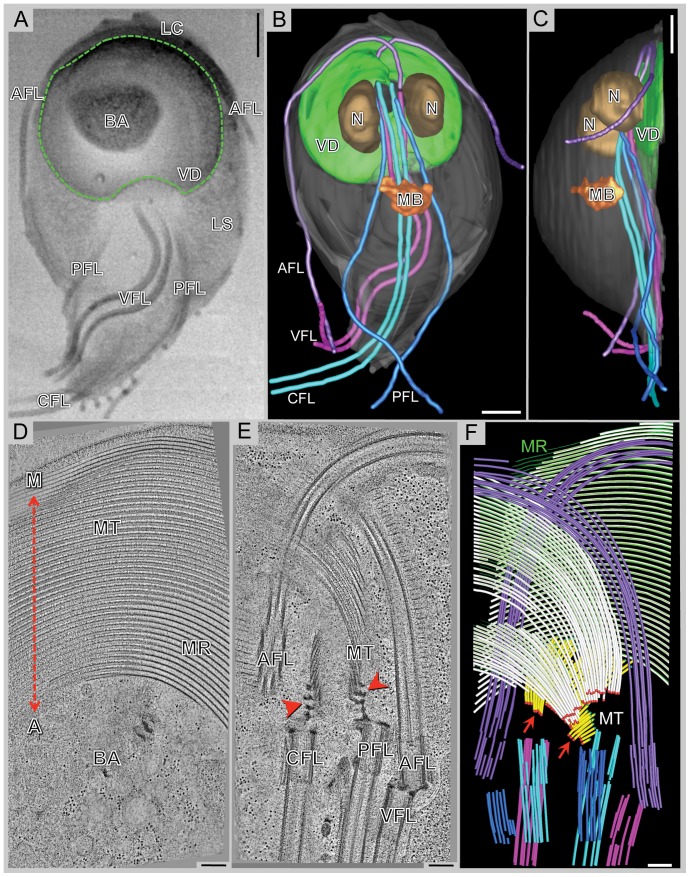
The complex microtubule cytoskeleton of *Giardia* reconstructed by 3View® and plastic-section tomography. **A**) Selected SEM slice (back-scattered electron signal) showing eight flagella [anterior flagella (AFL); caudal flagella (CFL); posterior-lateral flagella (PFL); and ventral flagella (VFL)], part of the ventral disc (VD: green outline), the bare area (BA), the lateral shield (LS), and lateral crest (LC). **B**) 3-D model of a whole-cell reconstruction: ventral disc, nucleus (N), median body (MB), and the four pairs of flagella. **C**) The side-view of the model shows that the entire microtubule cytoskeleton is located in the ventral part of the cell. **D**) 5 nm tomographic slice from a montaged, plastic serial section tomogram of a portion of the ventral disc. At the most ventral part of the disc, there are parallel microtubules and microribbons. The relationship of the disc to the helical axis is as indicated: margin-facing (M) or axis-facing (A). The bare area (BA) is also indicated. **E**) 5 nm tomographic slice showing the arrangement of four basal bodies and how the microtubules (MT) of the ventral disc originate from dense bands (arrows). **F**) Model from the tomographic reconstruction showing the supernumerary microtubules (yellow) are ventral to the ventral disc microtubules (white). Microtubule ends are classified as either capped (red dots, arrows) or open (green dots). Microribbons are shown in green. One of the anterior flagella (purple) penetrates the overlap zone. Scale bars in A–C = 2 µm, D–F = 200 nm.

The ventral disc of *Giardia lamblia* constitutes one example of such a highly regular microtubule-based array that maintains a strong extracellular attachment to the microvilli in the small intestine [8] that allows the parasite to colonize and resist peristaltic flow. The ventral disc is composed of a left-handed spiral array of parallel microtubules and tightly associated microribbons (Figure 1D) [9]–[12] that is surrounded by a fibrillar structure called the lateral crest (Figure 1A; LC). A region in the center of the ventral disc spiral that lacks microtubules, but instead features numerous vesicles, is termed the “bare area” (Figures 1A, 1D) [13]. 32 disc-associated proteins have been identified with a proteomics approach [14]. They were spatially analyzed in the cell by GFP-labeling and found to localize to the ventral disc and the lateral crest [14]. High-resolution structural details and interaction properties with ventral disc microtubules remains largely unknown. Likewise, the molecular mechanism by which the ventral disc mediates attachment is still unclear. In addition to the ventral disc, the interphase microtubule cytoskeleton of *Giardia* (Figure 1) also includes the median body (a structure of unknown function), four pairs of flagella, and the funis (not shown).

Attachment and detachment of *Giardia* trophozoites occurs within seconds in a series of steps characterized by defined contacts of the ventral disc region with surfaces. These contacts are readily visualized by live imaging of trophozoites with total internal reflection microscopy [15]. Distinct stages of attachment include trophozoite skimming on the surface followed by the formation of a seal between the attachment surface and disc via the lateral crest, then increased contact of the lateral shield region of the cell body with the surface, and finally increased contact of the bare area with the surface. During these stages of attachment, the ventral disc maintains a dome-shaped conformation and only the lateral crest and bare area region touch the surface [15]. Morpholino-based depletion of the abundant median body protein (originally found in the median body and therefore named as such) results in defects in the ventral disc, including a flattened disc structure and weakened attachment *in vitro* [16].

In the dynamic environment of the small intestine of the host, attachment site recognition, flagellar motility, and specific disc conformations contribute to parasite attachment to the intestinal microvilli. Flagellar motility has been proposed to create and maintain a negative pressure differential underneath the ventral disc [12]. However, more recent work suggests that flagellar motility is essential for positioning trophozoites, but is not required to maintain attachment *in vitro* [15]. The degree to which the disc substructure changes during attachment remains unknown, but conformational changes in the overall disc elements (microtubules, microribbons, etc.) are likely responsible for surface attachment. Such conformational changes may allow the cell to adhere to flat *in vitro* surfaces (i.e., glass) by a suction-type mechanism. *In vivo* attachment may include other mechanisms such as the disc “grasping” the intestinal epithelium. Both types of mechanisms would create a negative pressure differential underneath the disc [17].

With a limited understanding of ventral disc molecular composition, 3-D structure, and conformational dynamics during attachment, we are still in the early stages of defining the biophysical and molecular mechanisms by which *Giardia* attaches to the host. This comprehensive 3-D analysis of the ventral disc provides a structural model to explore how changes in disc microtubules and associated structures may enable overall conformational dynamics of the ventral disc during attachment and infection. Furthermore, this study underscores how highly organized microtubule-based arrays can evolve novel cellular functions in diverse eukaryotic cells. Despite previous work by Holberton [9], the *Giardia* ventral disc has never been described in three dimensions to such detail, and this study will now serve as a platform for further studies into the cellular functions of the unknown structures described within the disc.

## Results

**Figure 2:**
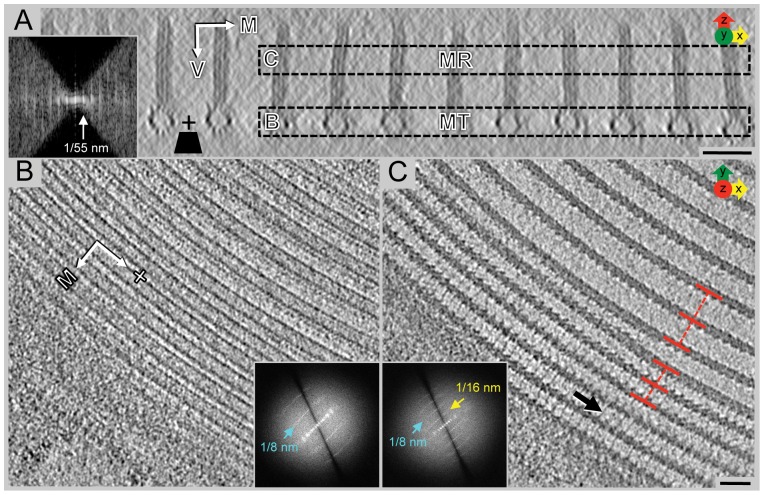
Cryo-electron tomography of the ventral disc. To describe the orientation, the dorsal-ventral (V) line and the axis-facing and margin-facing (M) sides of the microtubule are labeled. Microtubule polarity is indicated (+). **A**) 75 nm cryo-tomographic XZ-slice from Tomo-1 showing the ventral disc, an array of parallel microtubules with microribbons. The power spectrum (inset) shows the regular ∼55 nm spacing between neighboring microtubule-microribbon complexes. The missing wedge, a limitation inherited to tomographic data, is illustrated by the large, black, wedge-shaped area in the power spectrum. **B**) 25 nm tomographic XY-slice from box B in panel A. The power spectrum (inset) shows a repetitive unit every 8 nm. C) 25 nm tomographic XY-slice from box C in panel A. Microribbons have 16 nm repeats (power spectrum, inset). The lateral spacing of the microribbons (and underlying microtubules) is much closer at the margin of the disc (red lines). Scale bars, 50 nm.

**Figure 3:**
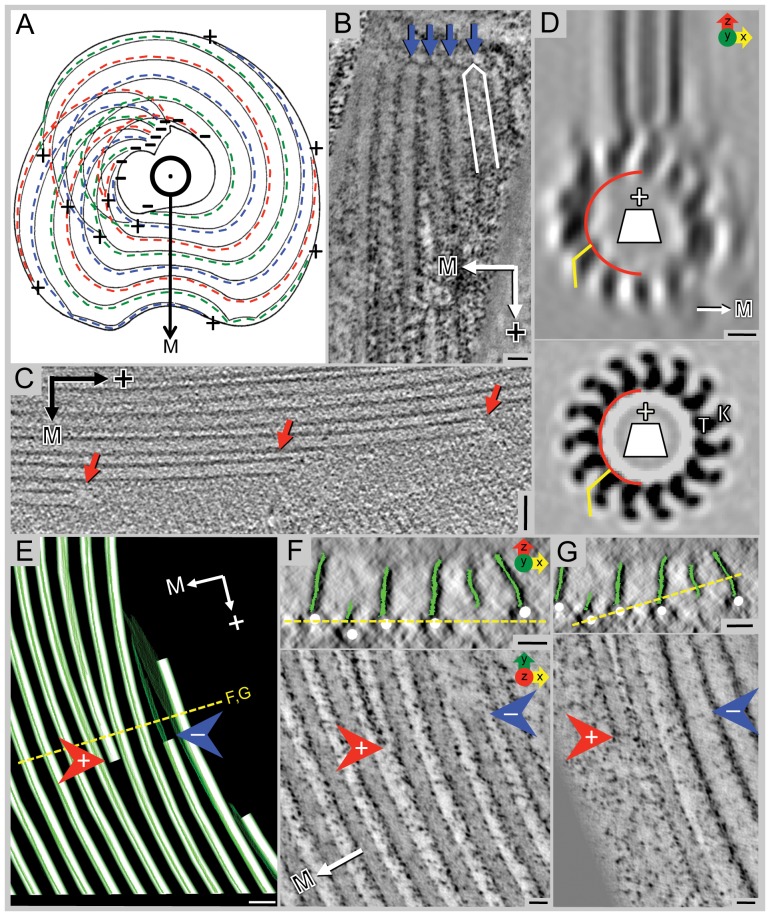
Polarity of ventral disc microtubules is unambiguous with minus-ends originating at dense bands or at the inside edge of the spiral. **A**) Sketch showing the ventral disc with the dorsal side pointing torward the viewer. Microtubules start with their minus-ends near the overlap zone and spiral downward to the ventral side, thereby forming a left-handed helix. The repetitive units are on the margin-facing side (→M) of the microtubules (dotted colored lines). **B**) A 10 nm plastic-tomographic slice shows capped microtubule ends (blue arrows) at the dense bands, indicating their minus-ends, while panel **C**) shows open microtubule ends (red arrows) at the periphery of the disc, typical for plus-ends. **D**) An end-on view from a helical reconstruction of a bovine microtubule decorated with kinesin-1 motor domains (K) (for example see [61]); when viewed from the minus-end, tubulin (T) shows a right-handed slew while globular microtubule-associated proteins (MAPs) such as kinesin motor domains often bend torward the left (lower panel). The same pattern is visible in the 3-D average of the tomographic reconstruction (upper panel), though with less clarity due to the missing wedge effects. **E**) A portion of the model of the plastic-section tomogram from Figure 1D showing a plus-end (red arrow) of a microtubule that is ending within the spiral and a minus-end (blue arrow) beginning at the inner edge of the spiral. The microribbons (green) of the inserted microtubules (white) are proximal to the minus-end (blue arrow) of the microtubule. **F, G**) The upper panels show the plastic-section tomogram in cross-section with the microribbons modeled in green and microtubules in white. The yellow line shows the line of rotation 90° to make the views in the lower panels. The microtubule ending within the spiral (red arrow) is slightly below the neighboring microtubules. The microtubule beginning at the inner edge of the spiral (blue arrow) starts above the neighboring microtubules. Scale bars in B and C = 50 nm; D = 5 nm; E = 50 nm; F and G = 25 nm. Panel A: adapted with permission from [11].

**Figure 4:**
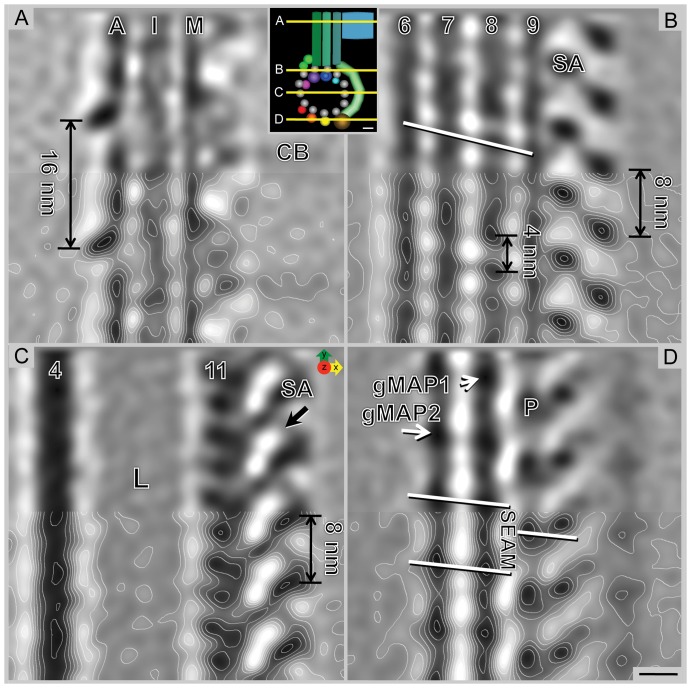
Grand average of the ventral disc microtubule-microribbon complex. Selected 0.776 nm XY-slices from the grand average (inset shows the location of each slice of the grand average). **A**) Microribbons are made of three sheets; axis-facing (A), inner sheet (I), and margin-facing (M). The overall microribbon structure shows a distinct 16 nm repeat over two successive αβ-tubulin dimers. Crossbridges (CB) are best visible on the margin-facing sheet but most likely contact the axis-facing sheet on the other side. **B**) Slice through the dorsal-facing microtubule wall near the microribbon attachment point showing the α- and β-tubulin densities and the regions of the side-arms (SA) forming an axial 8 nm repeat. The white line shows the characteristic pseudo-helix of neighboring protofilaments (PF6–9), which are composed of α- and β-tubulin subunits at a 4 nm spacing. **C**) The lumen (L) of the microtubule is empty. The associated side-arms are connected laterally (arrow). **D**) A slice through the most ventral microtubule-associated densities (gMAP1, gMAP2, and P) reveals the position of the microtubule seam—the offset is between gMAP1 and the paddle (P), but there is no offset between gMAP2 and gMAP1. Scale bar = 5 nm.

**Figure 5:**
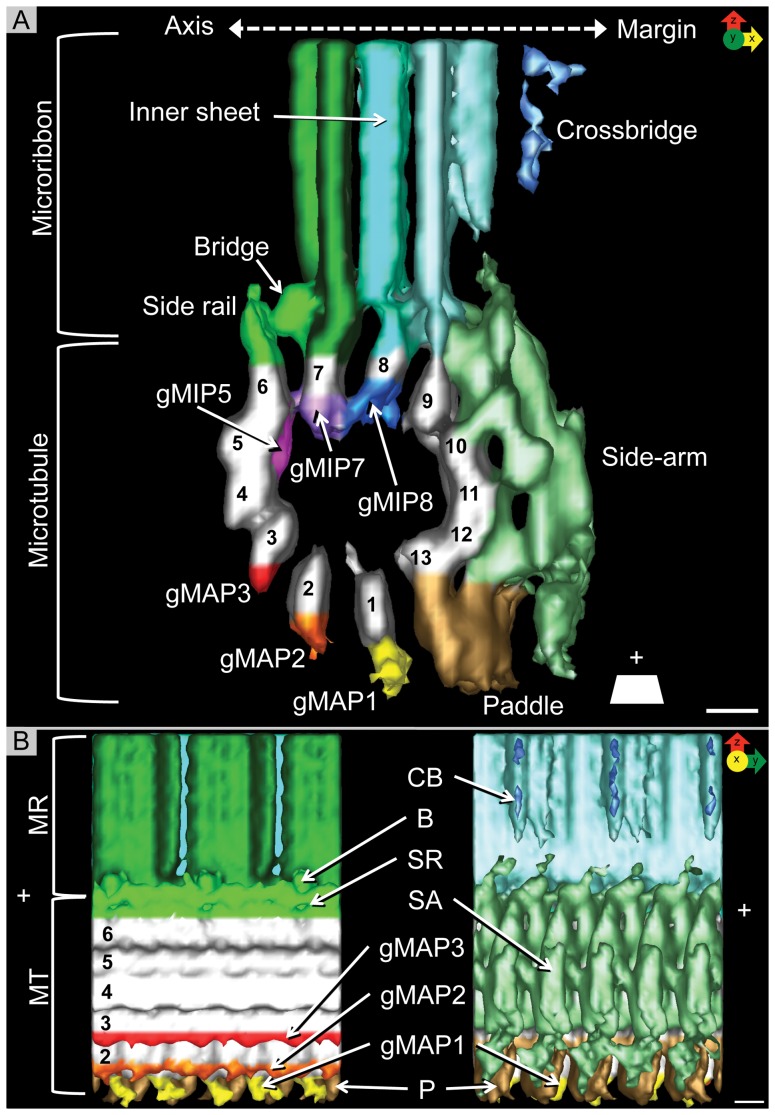
Isosurface representation of the grand average. **A**) View along the microtubule axis torward the plus-end. Each protofilament is numbered clockwise, starting with 1 at the location of the seam (see Figure 4D). Microribbons consist of three parallel sheets: axis-facing sheet, inner sheet, and margin-facing sheet. The crossbridges are visible on the margin-facing sheet. The axis-facing sheet is connected to the side rail, a fibrous structure attached to protofilament 6, via the bridge. There are several giardial microtubule inner proteins (gMIPs) associated with the inner wall of the microtubule on protofilaments 5, 7, and 8 (gMIP5, gMIP7, and gMIP8). There are also giardial microtubule-associated proteins (gMAPs) attached to the outer wall at protofilaments 1, 2, and 3 (gMAP1, gMAP2, and gMAP3). The side-arms (SA) span protofilaments 9–12 and are associated with the paddle (P), which is connected to protofilament 13. **B**) Axis-facing (left) and margin-facing (right) views. The axis-facing side has 2 “naked” protofilaments (PF4 and PF5). All three gMAPs (gMAP1, gMAP2, gMAP3) are visible as well as part of the paddle (P). The side rail (SR) on protofilament 6 is connected to the axis-facing sheet of the microribbon via the bridge (B). On the margin-facing side, gMAP1 is barely visible behind the paddle. The side-arm covers the rest of the microtubule. The crossbridges (CB) are evident on the margin-facing sheet (M) of the microribbon. Scale bars = 5 nm.

**Figure 6:**
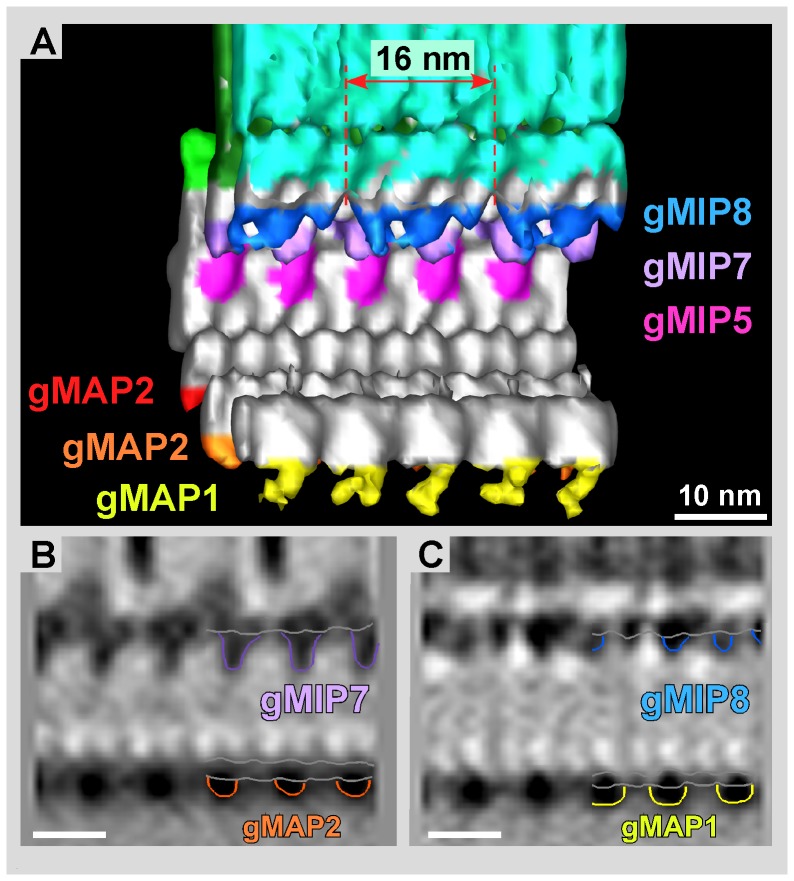
MIPs on the microtubule inner side of the grand average. **A**) Isosurface representation of the inner microtubule wall and associated gMIPs. While gMIP5 and gMIP7 appear regularly every 8 nm, according to the αβ-tubulin dimer repeat, gMIP8 clearly exhibits a 16 nm repeat that reaches over two consecutive dimers along protofilament 8. Panels B and C show vertical 0.776 nm slices through the volume in A, cutting through protofilament 7 (**B**) and protofilament 8 (**C**), respectively. Scale bars = 10 nm.

**Figure 7:**
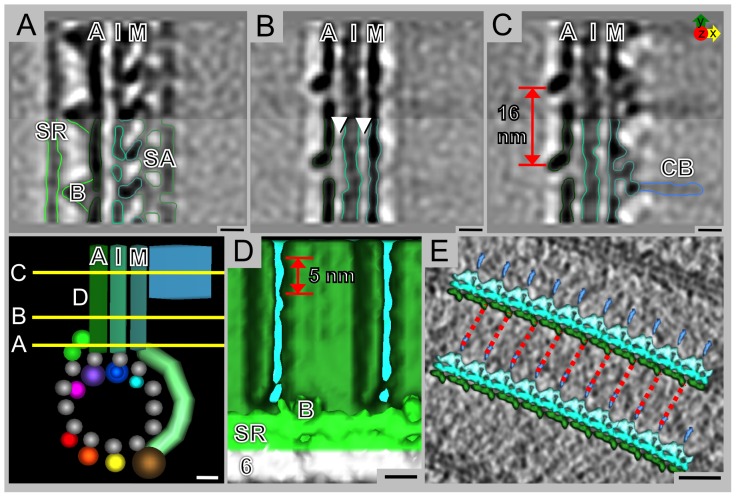
Microribbons of the ventral disc are composed of three parallel sheets. **A**) The bridge (B) connects the axis-facing sheet (A) to the side rail (SR). In addition, there is a complex array of proteins connecting the side-arm (SA) to the margin-facing sheet (M). The inner sheet (I) of the microribbon is partially associated with the margin-facing sheet. **B**) Lateral connections between the three sheets are evident (arrowheads). **C**) The axis-facing sheet has a distinct 16 nm repeat of 3 domains, one of which sticks out and is likely the attachment site of the crossbridges (CB) from the neighboring microribbon. **D**) A faint 5 nm repeat is evident along the dorsal-ventral line of the axis-facing sheet. **E**) The grand average isosurface has been inserted back into the original data, where each segment of the model has been rotated and shifted according to the parameters derived from the average. Dotted red lines have been drawn to indicate that the crossbridges on the margin-facing side likely connect to the 16 nm protrusions on the axis-facing side of the microribbon. Scale bars A–D, schematic = 5 nm, E = 25 nm.

The type of structural data obtained in this study is comprised of A) an overview of the microtubule cytoskeleton of the whole organism (Figures 1A–1C) at ∼75 nm resolution, B) the arrangement of the ventral disc microtubules/microribbons using conventional electron tomography (Figures 1D–1F) at ∼5 nm resolution, and C) unprecedented higher resolution (>3 nm) detail of microtubule and microribbon organization (Figures 2, 3, 4, 5, 6, 7) using volume-based averaging procedures on tomograms of frozen-hydrated specimens.

### Whole cell reconstruction of a *Giardia* trophozoite illustrates the highly structured 3-D architecture of the microtubule cytoskeleton

In addition to the ventral disc, *Giardia* trophozoites have a complex three-dimensional microtubule cytoskeleton, including eight flagella (anterior, caudal, posterior-lateral, and ventral) with cytoplasmic and membrane-bound regions, the median body, the funis, and the supernumerary microtubules. Here we have obtained the first 3-D reconstruction of an entire *Giardia lamblia* trophozoite using a new procedure called 3View® [18] that has been developed by Gatan Inc. (Pleasanton, CA). 3View® combines sequential microtome sectioning with scanning electron microscopy back-scattered electron imaging of each fresh blockface (Figure 1). This approach visualizes the 3-D relationships of the primary cytoskeletal elements of an attached trophozoite (Figures 1A–1C and Videos S1, S2). The overall dimensions of the cell in Figure 1 (A–C) are ∼10 µm×∼18 µm×∼7 µm (width, length, height). The total cell volume is ∼550 µm^3^. All four pairs of flagella originate from basal bodies that are located between the nuclei. All of them have cytoplasmic regions of variable lengths before exiting the cell as membrane-enclosed flagella, measuring as follows: ventral flagella at 4.6 µm, anterior flagella at 8.6 µm, posterior-lateral flagella at 9.0 µm, and caudal flagella at 14.8 µm. One anterior axoneme extends through the overlap zone of the ventral disc spiral. The bulk of the microtubule cytoskeleton is associated with the most ventral portion of the cell (Figure 1C), facing the host intestinal microvilli. The ventral disc is ∼8 µm in diameter and has a dome-shaped conformation that rises ∼2 µm from the surface when the cell is attached with the bare area in contact with the surface. The ventral disc represents about 2% of the total cell volume.

Our data of plastic-embedded trophozoites presented here now provides a tomographic 3-D structural analysis at ∼5 nm resolution (Video S3), improving on previous work by Holberton [9], [10]. The most dominant feature of the ventral disc is the highly organized array of evenly spaced parallel microtubules (Figures 1D, 2A, 2B), tightly linked to microribbons (Figures 1D, 2A, 2C). The ventral disc contains ∼50 microtubules, each of which can be up to 20 µm long [11]. The majority of the microtubule-microribbon complexes originates from a series of dense bands at a region near the caudal and posterior-lateral basal bodies (Figure 1E) and form a left-handed spiral (Figure 3A). Microribbons connect to the dorsal-facing surface of each microtubule and, depending on their location within the disc, extend between 150-400 nm into the cytosol (Figure 1F, 2A). The microribbons extend all the way into the overlap zone, the region where the beginning and end of the spiral overlaps (Figure 1F and Video S4; [16]). Occasionally, an array of microtubules called “supernumerary microtubules” [9], which lack associated microribbons and form a short right-handed spiral fragment (Figure 1F). Relative to the rotation axis of the microtubule spiral in the ventral disc, we describe microtubule-associated densities that are located toward the spiral's axis as “axis-facing” while structures facing away are called “margin-facing” (Figure 3A).

### Cryo-electron tomography reveals numerous regularly arranged densities on ventral disc microtubules that mirror the axial 8 nm repeat of the αβ-tubulin dimer

To explore the 3-D structure of the ventral disc in higher detail and determine the intimate contacts that hold adjacent microtubule-microribbon complexes together, we used cryo-electron tomography (cryo-ET) on intact, isolated cytoskeletons from trophozoites. Cryo-ET is the only method that allows 3-D analyses at molecular detail on such large and complex organelles as an intact ventral disc. Since whole trophozoites are far too thick (>5 µm in height) to be imaged directly by cryo-EM (the limit is about 500 nm), we isolated ventral discs and associated structures. In the most successful preparations, the ventral disc could be isolated with the eight basal bodies and flagella still associated and well preserved (Figure S1A). Once isolated, the ventral discs appeared flattened (∼9 µm in diameter X ∼1 µm thick), unlike the dome-shaped discs imaged *in vivo* (∼8 µm×∼2 µm). Co-isolated flagella tended to stay in the same positions as within intact cells, suggesting that they are kept in place by numerous molecular connectors between the basal bodies and ventral disc.

On areas near the outer edges of the disc that were thin enough for cryo-ET, we were able to collect cryo-tomograms to ∼2.8 nm resolution (Figures 2, S1C–S1G). We collected over 40 tomographic tilt-series from which we chose the best five datasets (named: Tomo-1—Tomo-5) for subsequent volume averaging [19], [20]. Figure 2A shows an example of a ventral disc cryo-tomogram (Tomo-1). The missing wedge of data occurs from tilt limitations of the electron microscope stage (±60 degrees instead of ±90 degrees) during tilt-series acquisition and is best visualized in Fourier space. In real-space reconstructions the most obvious effect is a non-isotropically resolved 3-D volume where the resolution along the Z-axis is about 1.5× lower than in the X and Y direction (Figures 2B, 2C; insets). However, depending on the orientation of the tilt-axis with respect to the XY-plane, there is anisotropy in this plane as well. The natural curvature of ventral disc microtubules allows the collection of data from discs at different orientations with respect to the tilt-axis (Figures S1C-S1G) that helps to reduce the missing wedge into a missing cone.

An end-on view of the microtubules and associated microribbon interface in the tomogram XZ-plane resolves microtubule protofilaments, microtubule-associated densities and details of the complex microribbon structure (Figure 2A). The XY-planes in Figures 2B and 2C cut through the ventral disc as indicated in Figure 2A (boxes marked B and C). The corresponding power spectrum within the microtubule range (inset in Figure 2B) reveals the characteristic 1/8 nm layer line reflections, which reflect the 8 nm αβ-tubulin dimer spacing along the microtubule. These repeats are present on the margin-facing side of the microtubule (Figures 2B, S1C–S1G). Microtubules do not appear to contain large, lumenal particle densities (Figure 4C). Power spectra on slices through the microribbons reveals layer line reflections at a 1/16 nm interval. Hence, the repeating motif of microribbons along the microtubule axis covers two consecutive αβ-tubulin dimers (Figures 2C, S1C–S1G). In many cases, we could identify connections between adjacent microribbons, called crossbridges [9], (Figures 2C, S1F). The lateral spacing between neighboring microtubule-microribbon complexes varies within the ventral disc. Those near the outer disc margin are much closer together (34 nm±2 nm) than those further inside (Figures 2B, 2C), which has an *in situ* spacing of 61 nm±2 nm (Figure 2A; inset).

### The ventral disc microtubule array has minus-ends at the basal bodies and blunt, open plus-ends at the margin

Due to the regular spiral arrangement of microtubules in the ventral disc, we unambiguously assigned microtubule polarity using three independent sets of observations in tomograms. First, in all cases the majority of microtubule-associated densities faced toward the outer margin, forming a very asymmetric decoration (Figures 2A, 2B and schematic in Figure 3A), suggesting that all disc microtubules share the same orientation. Second, our tomograms showed that the ends of microtubules embedded in the electron dense bands (Figure 1E) associated with the basal bodies were always capped (Figure 3B), indicating that these are minus-ends [21]. These capped ends are very close to each other, almost touching laterally. The opposite ends of microtubules at the ventral disc margins are blunt and open-ended (Figure 2C). Third, and probably the most convincing argument, averaged ventral disc microtubules viewed end-on, as seen in Figure 3D, revealed the typical protofilament slew that has been found on helically averaged kinesin-decorated microtubules on numerous occasions and which is an unambiguous marker for microtubule polarity [22], [23]. In summary, these observations are consistent with the disc microtubules being arranged with the capped minus-ends located near the basal bodies and the open plus-ends at the margin of the ventral disc.

### Ventral disc microtubule spacing is maintained by some microtubules ending within the spiral and new microtubules nucleating on the inner edge of the spiral

In order to maintain the regular spacing of the microtubule array within the ventral disc, microtubules end within the main spiral and new microtubules appear at the inner edge of the spiral that borders the bare area. Our tomographic data shows that microtubules ending within the spiral have blunt ends and sometimes the plus-end is found below the main body of the spiral (Figures 3E–3G; red arrow). Because these microtubules are not associated with the microtubule-organizing center (dense bands at basal bodies), they are blunt and open-ended.

### Novel molecular details of the microtubule-microribbon complex

Averaging of identical 2-D projections of repeating elements is a widely used method in electron microscopy to improve the signal-to-noise ratio for molecular data analysis in both two and three dimensions (i.e., single particle reconstruction, 2-D crystallography, random conical tilt reconstruction, and helical reconstruction [24]). However, none of those methods are applicable to large complex structures where averaging is not an option (i.e., organelles). Once a tomogram is completed, it may be possible to extract sub-volumes of potentially repetitive elements from the tomogram that may be further averaged for noise reduction. We have developed such a process in our lab called Particle Estimation for Electron Tomography, or PEET [19], [20]. With this software, we averaged a total of 7,719 individual sub-volumes from five different tomograms recorded on four different isolated ventral discs (Figure S1B). Each sub-volume was chosen based on a 16 nm repeat along the microtubule-microribbon axis (Figures S2A, S2B). We only selected sub-volumes from areas of the ventral disc that were neither part of the overlap zone nor near the very periphery of the disc. The final grand average (Figure 4 and Videos S5, S6) included 4,700 sub-volumes and resolved details to ∼2.8 nm resolution (50% Fourier-shell correlation criterion: Figure S2C). The averaged microtubule-microribbon 3-D map can be visualized in several ways. The distribution of internal densities within the 3-D map is best shown as contoured gray-levels on different slices through the map at various levels and orientations (Figures 4, 6B–6C, 7A–7C and Videos S5, S6). The full extent of the 3-D map, however, is best represented with an isosurface illustration showing the entire volume at one particular density threshold value that can be varied, but is typically chosen so that it presents most (90–95%) of the densities but without including too much unrelated noise (Figures 5, 6A, 7D and Video S7). A summary of the densities found in the grand average is listed in Table 1.

**Table 1:** List of separate structures found in the 3-D map.

Structure	Symbol	Repeat	Location
Protofilament	PF(1–13)	4 nm	Forms a 13-protofilament microtubule
Axis-facing sheet	A	3 domains in 16 nm	Microribbon, attaches to P7 at base
Inner sheet	I	16 nm	Microribbon, attaches to P8 at base
Margin-facing sheet	M	16 nm	Microribbon, attaches to P9 at base
Crossbridge	CB	16 nm	Connects A to M of adjacent microribbons
Side rail	SR	Fibrous (continuous)	Completely along outer surface of PF6
Bridge	B	16 nm	Connects SR to A
Side-arm	SA	8 nm	Spans PF9-PF12
Paddle	P	8 nm	PF13 (with PF1, location of seam)
Giardial microtubule-associated protein 1	gMAP1	8 nm	PF1 (with PF13, location of seam)
Giardial microtubule-associated protein 2	gMAP2	8 nm	PF2
Giardial microtubule-associated protein 3	gMAP3	8 nm	PF3
Giardial microtubule inner protein 5	gMIP5	8 nm	PF5
Giardial microtubule inner protein 7	gMIP7	8 nm	PF7
Giardial microtubule inner protein 8	gMIP8	3 domains in 8 nm	PF8

The averaging procedure was based on a 16 nm long repetitive volume along the microtubule axis that comprised two consecutive tubulin dimers. A single tubulin monomer is 4 nm, a single αβ-tubulin dimer is 8 nm, and two αβ-tubulin dimers are 16 nm.

Figure 4 shows single XY-slices of 0.776 nm thickness parallel to the microtubule plane descending from dorsal to ventral. Figure 4A cuts through the microribbon and reveals the 16 nm repeat. Faint signals for lateral crossbridges (CB) are seen at 16 nm intervals at the margin-facing (M) sheet. The axis-facing (A) sheet reveals three distinct axial density intervals over a total length of 16 nm. (Figure 4A). Figure 4B cuts through the dorsal edge of the microtubule, indicating the left-handed pseudo-helical pitch of a 13-protofilament microtubule. The 4 nm tubulin monomer repeat is visible in the microtubule wall, although at 2.8 nm resolution we cannot distinguish α-tubulin from β-tubulin, which are structurally very similar. The dorsal ends of the side-arms (SA) are evident, forming an 8 nm repeat, following the axial αβ-tubulin dimer repeat. A plane through the center of the microtubule reveals the mostly empty lumen (Figure 4C: L) and large side-arm densities (Figure 4C; SA) on the margin-facing side that maintain the axial 8 nm repeat and appear connected sideward along the microtubule axis (Figure 4C; arrow). Finally, Figure 4D slices through the ventral portion of the microtubule, revealing densities for two giardial microtubule-associated proteins (Figure 4D; gMAP1 and gMAP2), a distinct portion of the side-arm, which we call the “paddle” (Figure 4D; P), and the location of the microtubule seam.

### The microtubule lattice seam reproducibly locates to the same position in all ventral disc tomograms

In our 3-D map we were able to precisely locate the microtubule lattice seam (Figure 4D). This is quite remarkable as usually the seam cannot be located at this resolution due to the strong structural similarities between α- and β-tubulin. However, due to the repetitive nature of MAPs that mark the exact position of the αβ-tubulin dimers with respect to each other, the seam can be visualized (e.g. see [25]). The microtubule lattice seam is a characteristic feature of most microtubules found *in vivo* and, in particular, those composed of 12-, 13-, or 14-protofilaments. It marks the one lateral interaction between protofilaments where the B-lattice (lateral α-α or β-β interaction between adjacent protofilaments) is replaced by an A-lattice (α-β interaction) [26]; reviewed in [27]. Resolution of the microtubule seam in the averaged densities required the same microtubule orientation over different cells at the site of the microtubule seam. The lateral stagger of the left protofilament is 4.9 nm rather than 0.9 nm found in a B-lattice. This 4.9 nm lateral step between gMAPs unambiguously marks an A-lattice interaction and hence the location of the tubulin lattice seam [28].

### Ventral disc microtubules feature giardial microtubule inner proteins (gMIPs) as well as giardial microtubule-associated proteins (gMAPs)

Isosurface representations of the grand average (Figure 5A; axial view and Figure 5B; lateral views and Figure 6A; cutaway view and Video S7) provide an overall view of the 3-D arrangement of the microtubule-microribbon complex and associated proteins. The microtubule is composed of 13 protofilaments, numbered in a clockwise direction from the seam (Figure 5A). Unless otherwise noted, in all subsequent figures, the structure will be shown with the margin-facing side of the microtubule to the right, the ventral-facing side down, and the microtubule minus-end torward the viewer. Each structure of the grand average is color-coded throughout all figures.

Apart from the microribbons, ventral disc microtubules show multiple protruding densities that represent novel associated proteinaceous complexes, mostly bound to the outer microtubule wall, but some densities are also attached to the inner microtubule wall. The inner microtubule densities are located on protofilaments PF5, PF7, and PF8. Here we have named them giardial microtubule inner protein (gMIPs: gMIP5, gMIP7, and gMIP8) corresponding to the protofilament number to which each is attached (Figures 5A, 6, S3A–S3E). While gMIP5 (Figures 5A, S3A, S3E) has an 8 nm repeat relative to the protofilament and is globular, it seems to span the entire distance between PF4 and PF6 and could be attached to PF4 and PF6 as well (Figure 6A). The density for gMIP7 (Figures 5A, 6A, S3B, S3D, S3E) has a columnar shape that extends into the lumen of the microtubule and presumably binds between the α- and β- subunits, repeating every 8 nm. Interestingly, gMIP8 (Figures 5, 6A, 6C, S3C, S3D) consists of three smaller globular domains and repeats every 16 nm (Figure 6A, 6C). There may be a link between gMIP7 and gMIP8 (Figures 5A, S3D).

Ventral disc microtubules also have several giardial microtubule-associated protein complexes (gMAPs: gMAP1, gMAP2, and gMAP3) corresponding to densities found on the outside of the microtubule wall at PF1, PF2, and PF3, respectively. While the inner densities may correspond to a single polypeptide, the outer densities appear to be large complex assemblies of multiple proteins. However, for simplicity we still name them gMAPs, knowing that a gMAP marked here as a single unit may indeed be composed of multiple protein domains. In our grand average all three gMAPs appear to repeat axially every 8 nm. All gMAPs are mostly globular (Figures 5A, 5B, S3A–S3C, S3I) and are found on the ventral-facing side of the microtubule—nearest the plasma membrane.

The axis-facing side of ventral disc microtubules is essentially bare, lacking any detectable microtubule-associated proteins on PF4 and PF5, with PF4 being the only protofilament with no detectable associated proteins (either gMAP or gMIP) (Figure 5A). The margin-facing side of the microtubules are decorated by large, complex, repeating densities, called side-arms [9] that are composed of an as-yet-unknown assembly of proteins. The full extent of these side-arms (SA) span PF9–PF12 with an additional structure, the paddle (P), attached to PF13 (Figures 5, S3F–S3M). Side-arms repeat axially every 8 nm and are connected to adjacent side-arms laterally where they attach to the microtubule wall at PF10 (Figures S3G, S3M). The side-arm structure changes continuously with corresponding changes in the pseudo-helix of the microtubule wall. Specifically, a region of the side-arms is attached to the outer wall of the microtubule from PF9–PF12 continuously (Figures S3J–S3M) and other parts extend about 10 nm from the outer surface of the microtubule wall (Figures S3F–S3I).

### Microribbons are tri-laminar and are connected laterally by flexible crossbridges

Microribbons have three distinct but closely apposed sheets. The axis-facing sheet attaches to the side rail (SR), a fibrous density covering PF6, every 16 nm via the bridge (B: in Figures 5, 7A, S3B) and is directly attached to PF7 at its base (Figure 5A). The axis-facing sheet shows a distinct 16 nm lateral repeat along its entire height (Figures 5, 7B, 7C), which appears to be composed of three major domains. The inner sheet attaches to PF8 (Figure 5A) and appears to be connected to both outer sheets (Figure 7B). The margin-facing sheet is attached to the inner sheet, the side-arms, and PF9 at its base (Figure 5A). There is a repetitive structural motif that reaches over two axial tubulin dimers, which is evident on both the axis-facing and margin-facing sheets (Figures 2C, 4A, 7A–7D, S1C–S1G). Hence, there is an apparent mismatch between the sheet structure and individual tubulin dimers. The axially repeating densities of the inner- and margin-facing sheets are less pronounced (Figure 4A). In addition to the 16 nm axial repeat there is a faint dorsal-ventral striation with a repeat of approximately 5 nm (Figure 7D), also shown by Holberton [10].

Microribbons near the edge of the ventral disc typically extend 100 nm into the cell body and are connected laterally by crossbridges, which are found ∼10 nm dorsal to the microtubule-microribbon interface every 16 nm (Figures 2C, 7C, S1F). Figure 7E shows the crossbridges of the margin-facing sheet of one microribbon have a trajectory that extends to the domain that sticks out the farthest on the axis-facing sheet of the neighboring microribbon.

## Discussion

Here we report on a detailed 3-D analysis of the ventral disc of *Giardia* trophozoites, which serves three general scientific aspects that are potentially very relevant for future studies that will go beyond the sole description of a unique biological organelle. First, it provides a basis for a thorough identification of the various components that we found here. It will help enhance our knowledge on the complex issues regarding microtubule dynamics and hyper-stabilization by associated factors, and possibly discover novel molecular mechanisms for microtubule-associated complexes. Secondly, further insight into the function of the ventral disc will connect molecular structural detail with the physical properties of microvilli attachment of *Giardia* during host infection. And lastly, a detailed characterization of the components involved in this organelle that has no equivalent in any metazoan organism might lead to *Giardia*-specific drug targets that can be applied very selectively with high therapeutic efficiency and least possible side effects for patients.

### Whole-cell 3-D data at intermediate resolution reveals the detailed cytoskeletal architecture of the trophozoite

Figures 1A–1C show the results of a novel 3-D imaging approach, 3View® ([18], Gatan, Inc. Pleasanton, CA), that combines microtomy on plastic-embedded, intact *Giardia* trophozoites with scanning electron microscopy (SEM), where the exposed surface after each microtome section is imaged and mounted into a 3-D volume with a resolution of ∼75 nm in all three dimensions. This has allowed us to map out the microtubule cytoskeleton in a wild type trophozoite, confirming reports about flagella placement [29] previously visualized by light microscopy. We also gain a sense of the overall shape of the ventral disc with respect to the overall cellular architecture (Figure 1).

Greatly improved spatial 3-D resolution (∼5 nm) is achieved for large volumes by stacking and aligning tomograms from consecutive sections of plastic-embedded cells (Figures 1D–1F). However, the technique is time-intensive and despite the stacking of three montaged tomograms, it still reveals a much smaller field of view and volume (∼11 µm^3^) than that of an entire disc (∼54 µm^3^). In the reconstruction presented in Figures 1D–1F we looked at the part of the disc that encloses the beginning of the microtubule helix, part of the overlap zone, and the area between the two nuclei that houses the basal bodies. We can see that the parallel array of microtubules originate from dense bands associated with the caudal and posterior-lateral basal bodies (Figure 1E). Closer investigations of the microtubule ends in these tomograms show that the ends found in the dense bands (microtubule organizing centers) are capped (Figure 3B) while the other ends are open (Figure 3C). These features are reliable indicators for the microtubule polarity in the ventral disc.

Throughout the majority of the disc, the neighboring microtubule-microribbon complexes maintain a constant lateral spacing (Figures 2A, S1C–S1G), reminiscent of the subpellicular microtubule array of *Trypanosoma brucei* [30], which has microtubules inserted within the array at varying intervals. As microtubules end within the ventral disc spiral, new microtubules are added at the inner edge near the bare area of the spiral. When these microtubules are inserted at the inner edge, the microribbons are formed proximal (Figures 3E–3G; blue arrow) to the microtubule minus-end at some distance distal to the capped ends (Figures 3E–3G; red arrow). However, to fully understand how the spacing within the spiral is maintained, large area tomography or dual-beam tomography would be needed.

### The presence of giardial microtubule inner proteins (gMIPs) in the ventral disc may add to the hyperstability of the disc microtubule array and the connection to the sheets

We localized several giardial microtubule associated proteins (gMAPs) that are associated with the outer wall of the microtubule, and giardial microtubule inner proteins (gMIPs) that are associated with the inner wall of microtubules. Typically MAPs can both regulate the stability as well as the dynamics of microtubules. While known MAPs associate with the outer wall of diverse microtubules, MAPs found on the microtubule inner surface, or MIPs, have only been described in axonemal and subpellicular microtubules [5], [19], [31]–[33], and are distinct from microtubule luminal particles found in cytoplasmic and nuclear microtubules [34], [35]. Luminal particles are large enough to be directly visible in cryo-tomograms without the need for averaging techniques, unlike gMIPs, which are not obvious in the cryo-tomograms presented here. Luminal particles are not present in ventral disc microtubules of *Giardia* (Figures 2A, 2B, 3C, S1C–S1G).

There are no reports yet of MIPs found in cytoplasmic or nuclear microtubules. While in most cases the identity of MIPs remains unclear, Sui and Downing [32] identified a likely MIP candidate, tektin, a filamentous MIP found in the A-tubule of sea urchin axonemes. Unlike tektin, none of the gMIPs we found seem to be filamentous in nature, but feature distinct globular domains. Instead they more closely resemble MIP1 and MIP2 from the A-tubule and MIP3 found in the B-tubule of both *Chlamydomonas* and sea urchin axonemes [19], [33]. In particular, gMIP8, with three globular domains over a 16 nm repeat (Figure 6A, 6C) is similar to *Chlamydomonas* and sea urchin MIP3 [33]. Subpellicular microtubules of apicomplexan *Plasmodium* parasites contain a MIP that follows the axial 8 nm tubulin repeat [5]. MIPs seem predominant in microtubules that require added stability such as microtubules exposed to extreme bending forces as in flagellar axonemes [36] and hyperstable subpellicular microtubule arrays in apicomplexan parasites [5]. The position of the gMIPs on the inner wall of the microtubule at the microtubule-microribbon interface (PF5, PF7, PF8) may indicate a higher need for stabilization in this region, possibly supporting the association of the microribbons.

FRAP experiments with disc proteins [14] support the idea that the ventral disc is a hyperstable organelle. Since ventral disc microtubules also have MIPs, they may experience strain and/or other forces, suggesting that they may move or bend substantially during substrate attachment. In fact, the bending of the spiral during ventral disc assembly may require the presence of MIPs to stabilize the microtubules in a curved conformation and help maintain that curvature during the trophozoite's life cycle.

### Giardial microtubule-associated proteins (gMAPs) of unknown composition form a dense coat around the ventral disc microtubules

A large number of proteins and complexes associate with ventral disc microtubules and form a dense proteinacous coat, in particular on their margin-facing surface. It has been noted that many antibodies directed against tubulin in the ventral disc do not reach their epitope [14], [37]. Our data from the averaged ventral disc microtubules make it clear why this is the case; only two (PF4 and PF5) of the 13 protofilaments are not covered by gMAPs (Figure 5). Apart from the microribbons, the largest complexes are the very dominant side-arms (Figures 4B, 4C, 5). These were first described by Holberton [10] on tannic acid stained thin-sections. Holberton found that the side-arms were always located on the margin-facing side of the microtubule, an observation confirmed from our cryo-tomograms. We extend those observations by noting that the side-arms form a multi-protein complex that reaches laterally over at least four adjacent protofilaments (Figure 5A; PF9–PF12) and that may have different interaction sites on each of the four protofilament surfaces.

To date, proteomics helped to identify over 30 disc-associated proteins [14], [38]–[46] but this data provides few indications on the precise location of these proteins. Disc-associated proteins were initially termed “giardins”, with three separate gene families of giardins localizing to the ventral disc: three annexins, or α-giardins [38]–[41]; three striated fiber (SF)–assembling homologs, including β-giardin [42], [43], δ-giardin, SALP-1 [46]; and one novel protein, γ-giardin [44]. Nevertheless, the exact composition of the various microtubule-associated protein complexes observed here (microribbons, crossbridges, side-arms, paddles, etc.) and how each structural element contributes to the overall conformational dynamics of the ventral disc during attachment remains unclear. Hence, our detailed structural analysis does provide a starting point for developing models regarding the molecular mechanisms of attachment and provides a map where individual components may be filled in upon identification and localization by various methods.

### Microribbons are unique, stable microtubule-associated structures with an unknown function in disc conformational dynamics

Microribbons are trilaminar structures that extend in the dorsal direction from the ventral disc microtubules into the cytoplasm. The microribbons connect laterally via flexible crossbridges. They are comprised of several homologs of striated fiber (SF) assemblins, including β-giardin, δ-giardin, and SALP-1 [46]. Non-contractile SF-assemblins are the major component of striated microtubule-associated fibers (SMAFs) in flagellated green algae [47]. Microribbon-associated proteins are likely assembled into the ventral disc prior to cell division and do not appear to be dynamic or frequently exchanged. None of them have been shown to recover from photo-bleaching [14]. As with the functioning of SMAFs in the basal body apparatus of *Chlamydomonas* [47], it is likely that the microribbons provide an overall rigidity to the ventral disc.

Previously, the most extensive structural studies on microribbons were conducted by Holberton [9]–[11] who described crossbridges that occur axially at regular intervals of 15 nm [10]. We are certain that this 15 nm repeat most likely constitutes the same repeat our data shows to be 16 nm, actually matching the repeat of two consecutive αβ-tubulin dimers. There are two observations that argue against a 15 nm periodicity with a mismatch to the underlying microtubule periodicity. First, power spectra of ventral disc microribbon areas show a clear 16 nm layer line and, second, volume averaging produces the crossbridges at regular intervals. A mismatch would eliminate their densities through the averaging process. Holberton's ultrastructural data was collected from negative stained samples, which are prone to shrinkage effects, whereas our data was collected from microribbons in a frozen-hydrated state, representing the most near-to-native structure [48], [49]. Holberton also described a 4 nm periodic structure on the dorsal-ventral line of both axis-facing and margin-facing sheets [10]. We find a faint repeat in the dorsal-ventral direction of ∼5 nm. This difference may also come from the different specimen preparation methods or calibration errors.

### Crossbridges likely maintain lateral spacing and contribute to the overall ventral disc conformation

The lateral spacing between adjacent microtubule-microribbon complexes is much narrower at the very margin of the ventral disc (compare Figures 2B and 2C, lower left corner of panel with upper right corner of panel). In both cases crossbridges can be seen, albeit of different lengths. Hence, the variations in lateral spacings may be mediated by contractible crossbridges that involves them in the overall conformational dynamics of the ventral disc during attachment to the host. The entire ventral disc has been proposed to generate a negative pressure differential via its domed shape wherein the outer rim of the disc, or even the entire disc, contracts or relaxes [15]. This is believed to represent a contracted state relevant for intestinal attachment either working directly to generate suction-based forces for attachment [17], or via a grabbing or clutching mechanism [50].

The domed shape of the ventral disc is necessary to form proper surface contacts and maintain attachment and is mediated in part by the median body protein. Depletion of median body protein results in flattened discs lacking the overlap zone [16]. Crossbridges are also important for maintaining the dome shaped nature of the ventral disc. As the number of broken crossbridges increases, the distinctive dome is disrupted [11]. In our isolated cytoskeletons, the dome-shape has been lost and crossbridges are often hard to identify (Figures S1C–S1G). Crossbridges are particularly visible when they are shortened in areas where the lateral microtubule-microribbon spacing is narrow (Figure 2C). In general, crossbridges seem to be somewhat flexible in these preparations, explaining why they are smeared out in the grand average.

## Conclusions

Unlike for *in vitro* studies that are typically carried out on a few, isolated components, an intact cell or large organelle such as the ventral disc in *Giardia* combines numerous structures in a largely unknown 3-D arrangement. Some structures like microtubules are easily recognized based solely on shape or cellular location; others can only be identified with specific labels. It will take substantial effort to correlate the data available from fluorescence light-microscopy labeling of specific disc-associated proteins [14] with the electron microscopy structures presented here. One way of correlating the known and novel disc-associated proteins with the microtubule-associated ventral disc structures will be by using clonable high-electron dense labels such as metallothionein [51], [52] or metal-clustering peptides [53]. From our study, it is evident that identifying the specific proteins and proteinaceous complexes in the context of an intact cell or large and complex cellular organelle will continue to be a serious challenge in the future, even in a highly ordered structure such as the *Giardia* ventral disc. Nonetheless, this analysis provides a structural foundation for further proteomic studies into *Giardia*-specific protein complexes that later might provide a target for specific anti-*Giardia* drugs and therapies. Drugs affecting ventral disc structure, disc biogenesis, or disc conformational dynamics may directly or indirectly decrease trophozoite attachment in the host, and thus limit the initiation or extent of infection.

## Materials and Methods

All electron microscopy reagents were purchased from EMS (Hatfield, PA) unless otherwise noted.

### Cell culture

Wild type *Giardia lamblia* trophozoite cells (strain WBC6) were grown in modified TYI-S-33 media at 37°C [54]. Cultures were grown in 16 ml plastic screw-cap tubes to maintain an anaerobic environment.

### Serial block face scanning electron microscopy

Cells were plated on carbon-coated sapphire discs (Rudolf Brügger SA, Minuso Switzerland) in a homemade anaerobic chamber and allowed to attach for one hour. Cells were processed as described in [55], but modified as follows. Cells were first fixed with 2% gluteraldehyde in 0.05 M sodium cacodylate buffer for 30 minutes at room temperature and then rinsed 3× with buffer. A second fix of 2% OsO_4_, 0.8% K_3_Fe(CN)_6_ in buffer was added for 1 hour on ice and then rinsed 3× with buffer. A third fix of 0.15% tannic acid in buffer was added for 15 minutes at room temperature and rinsed 5× in buffer. A fourth fix of 2% OsO_4_ in buffer was added for 30 minutes and then rinsed 3× in dH_2_O. The sample was then dehydrated into acetone (25%, 50%, 75%, 90%, 95%, 3×100%, 5 minutes each) and flat-embedded in hard Epon over 3 days. 50% HF acid was used to dissolve away the Teflon-coated glass slides used in flat embedding. Blocks were then trimmed and placed into a Quanta 600 environmental scanning electron microscope (FEI-Company Inc., Eindhoven, The Netherlands) operating at 2.5 kV and 0.25 torr. The microscope was equipped with a diamond knife microtome (Gatan 3View®; Gatan Inc. Pleasanton, CA [18]). Serial 70 nm sections were cut and the block-face was imaged with backscatter detection. XY resolution is estimated at 75 nm. The stack of images was processed using Digital Micrograph (Gatan Inc., Pleasanton, CA), converted to MRC format, filtered, and modeled using IMOD [56].

### Room temperature tomography

Cells were plated onto carbon-coated sapphire discs (reviewed in [57]) as previously described and high pressure frozen using a BalTec HPM-010 (Leica, Vienna, Austria). The vitrified cells on discs were freeze-substituted in 4% OsO_4_ and 1% uranyl acetate in acetone and warmed from −90°C to room temperature. Discs were then embedded in Epon/Araldite and 300 nm sections were generated. 10 nm colloidal gold (Ted Pella, Redding, CA) was added for fiducial markers. Dual-axis serial section montaged tomograms were acquired with a Tecnai TF30 300 kV FEG (FEI-Company, Hillsboro, OR, and Eindhoven, The Netherlands) transmission electron microscope using SerialEM [58] from ±60° with 1° increments. R-weighted back projection tomograms were computed using IMOD [56]. Portions of the model were generated using IMOD and Shape [59].

### Cytoskeletal preparation

Cytoskeletons were isolated using a procedure modified from [11]. Cells grown in 16 ml screw-cap culture tubes were rinsed with 37°C PHEM buffer (60 mM PIPES, 25 mM HEPES, 10 mM EGTA, 2 mM MgCl_2_) to remove unattached and dead cells. 2 ml of fresh PHEM buffer with 2% triton-X-100 was added to the cells and transferred to an Eppendorf tube. Cytoskeletons were vortexed on low for 30 minutes, water bath sonicated for 1 minute and then checked in the light microscope. The vortex/sonication procedure was repeated until the cytoskeletons were adequately extracted. They were then rinsed 3× with PHEM buffer. A 4 µl drop of cytoskeletal prep was adsorbed onto a holey carbon grid (Quantifoil, Jena, Germany) and 1 µl of 10 nm colloidal gold was pipetted into the drop. The grids were hand-blotted and plunge-frozen into liquid ethane. Grids were maintained at LN_2_ temperatures until visualized by cryo-electron microscopy.

### Cryo-tomography

Grids with vitreous cytoskeletons were imaged using a Tecnai TF30. Images were obtained through a Tridiem Gatan Imaging Filter (Gatan Inc., Pleasanton, CA) operating with a slit width of 20 eV onto the Ultracam, a 4K lens-coupled CCD camera (Gatan Inc., Pleasanton, CA). Cryo-tilt-series were acquired using the automated tilt-series acquisition software, SerialEM [58]. A typical tilt-series spanned 120°, with 2° tilting increments and a pixel size of 0.776 nm. Defocus was either −6 µm or −4 µm. Tomograms were calculated with R-weighted back projection using IMOD [56], the contrast transfer function (CTF) was corrected [60] and the gold erased. A total of ∼40 tomograms were generated.

### 3-D volume averaging

We used our program, PEET (Particle Estimation for Electron Tomography; [19], [20]), to average 16 nm intervals along the axial length of the ventral disc microtubules. Five tomograms from four ventral discs were selected based on overall tomogram quality. Sub-volumes were selected by placing a model point every 16 nm at the microtubule/microribbon interface. We used PEET to remove duplicate particles and apply missing wedge compensation. We chose a single sub-volume from a tomogram as an initial reference and then averaged all of the tomograms together. For analysis, we calculated FSC curves to estimate resolution using the 50% criterion and used IMOD [56] to visualize the averages and 3-D models. To test for reference bias, we computed 5 different grand averages by using a sub-volume from each tomogram as a reference. In all cases, the grand averages were virtually identical.

## Supporting Information

Figure S1 **Cryo-electron tomography of ventral discs.** **A**) Isolated cytoskeleton with the ventral disc (VD) and all eight flagella (AFL, CFL, PFL, VFL) present. Areas suitable for cryo-tomography are over the hole in the carbon (box). **B**) A schematic representation of the ventral disc showing the location of each tomogram used in this study (1–5). Adapted with permission from [11]. **C–G**) Tomographic slices from each of the tilt-series used to generate the grand average (C, Tomo-1; D, Tomo-2; E, Tomo-3; F, Tomo-4; G, Tomo-5). The left panel is a 25 nm slice through the microtubules and the right panel is a 50 nm slice through the microribbons. Each tomogram is shown with its original orientation with the tilt-axis vertical. In all cases, the 8 nm repeat on the microtubule is obvious (arrow in F, left panel), but the crossbridges between adjacent microribbons are only sometimes seen clearly (arrow in F, right panel). Plus-end and margin directions are indicated. Scale bars in A = 2 µm, C–G = 100 nm.
(TIF)

Figure S2 **Sub-volume averaging.** **A, B**) Subvolumes were chosen every 16 nm along the axis of the microtubule at the microtubule/microribbon interface. **C**) Fourier-shell correlation of each individual tomogram average (Tomo-1—Tomo-5) and the grand average. Each number in parentheses shows the number of subvolumes used to calculate the Fourier-shell correlation. The grand average has a resolution of ∼28 Å.
(TIF)

Figure S3 **Giardial Microtubule Inner Proteins (gMIPs) and Giardial Microtubule-Associated Proteins (gMAPs) are key features of ventral disc microtubules.** **A**) gMIP5 (magenta) has an 8 nm repeat and is found on the inside surface of protofilament 5 (PF5), spanning the distance between PF4 and PF6. gMAP3 (red) occurs every 8 nm on PF3. **B**) gMIP7 (purple) is attached to PF7 every 8 nm. Part of the bridge (B; green) is attached to the outside surface of PF7. gMAP2 (orange) occurs every 8 nm and is attached to PF2. **C**) gMIP8 (blue) is attached to PF8 and has a 16 nm repeat of three globular domains (2 are shown in this slice). gMAP1 (yellow) is found every 8 nm and is attached to PF1. **D**) In the XY orientation, it is clear that gMIP8 has a 16 nm repeat consisting of three different densities. There is a possible lateral connection between gMIP7 and gMIP8 (arrowhead). **E**) Two of the three gMIPs are shown. gMIP5 (magenta) has an 8 nm repeat and is found on the inside surface of PF5. gMIP7 (purple) has an 8 nm repeat and is found on PF7. **F**) Side-arms follow an 8 nm repeat. Near the top of the side-arm, a portion is attached to PF9. **G**) The side-arm is attached to PF10 and has lateral connections between neighboring side-arms (arrow). **H**) Side-arms are attached to PF12. **I**) A portion of the side-arm has been differentiated as the paddle (brown). The seam of the microtubule is located between gMAP1 and the paddle (arrowhead). **J–M**) YZ-slices showing how the side-arms (SA) follow the helix of the microtubule. (**J**) Side-arms attach to the microtubule at PF10 and (**K**) PF12. The paddle attaches to PF13. (**L**) The beginning of the cross-bridges where they are attached to the marginal-facing sheet. (**M**) The same linker as in **G** between adjacent side-arms (arrow). Scale bars, 5 nm.
(TIF)

Video S1 **Whole-cell reconstruction of an attached** ***Giardia intestinalis*** **trophozoite using 3View®.** Each slice was obtained using a microtome inside a scanning electron microscope. After each section was removed, a backscatter-signal scanning electron micrograph was recorded [18]. IMOD [56] was used to model important features of the cytoskeleton and attachment sites. Each slice is 70 nm. The majority of organelles are visible with this method: Plasma membrane (grey), median body (orange), nuclei (brown), ventral disc (green), anterior flagella (purple), caudal flagella (cyan), posterior-lateral flagella (blue), and ventral flagella (magenta). Near the ventral portion of the cell, important components of attachment are seen (bare area, lateral crest, lateral shield). Scale bar, 2 µm.
(MOV)

Video S2 **Whole-cell reconstruction model showing relationships between cytoskeletal elements.** The Video starts with the raw data, then transitions into the modeled data created by IMOD [56]. The ventral portion of the cell contains most of the cytoskeletal elements—the ventral disc (VD), median body (MB), and 4 pairs of flagella (anterior flagella, AFL; caudal flagella, CFL; posterior-lateral flagella, PFL; ventral flagella, VFL) as well as the two nuclei (N). The bulk of the cytoskeletal elements are at the ventral portion of the cell—the attachment site to the host microvili. Scale bar, 2 µm.
(MOV)

Video S3 **Tomographic reconstruction of 3 serial-montaged sections of a** ***Giardia*** **trophozoite.** Each slice is 3 nm in the Z-plane. The transition zone between sections looks like a jump. Anterior flagella, AFL (purple); caudal flagella, CFL (cyan); posterior-lateral flagella, PFL (blue); ventral disc microtubules, MT (white); microribbons, MR (green); dense bands, DB; supernumerary microtubules, SMT (yellow). Margin-facing and axis-facing sides are shown for orientation. This volume is about ∼11 µm^3^ of the entire disc, which has a volume of ∼54 µm^3^. Scale bar, 500 nm.
(MOV)

Video S4 **Model of tomographic reconstruction of a** ***Giardia*** **trophozoite.** The model was generated using IMOD [56]. The movie starts with the viewer looking torward the ventral surface of the cell. Major cytoskeletal components are present: anterior flagella, AFL (purple); caudal flagella, CFL (cyan); posterior-lateral flagella, PFL (blue); ventral flagella, VFL (magenta); supernumerary microtubules, SMT (yellow); microribbons, MR (green); microtubules, MT (white); open ends are green spheres; closed (capped) ends are red spheres. The overlap zone and dorsal-ventral line are indicated. The movie ends with the viewer looking torward the dorsal surface of the cell. Scale bar, 500 nm.
(MOV)

Video S5 **Grand average of 4700 subvolumes by the 16 nm axial repeat at the microtubule/microribbon interface (XY-plane).** Each step is 0.776 nm. The grand average generated by the software PEET [19], [20] shows the structures associated with ventral disc microtubules. Orientation markers are shown. The movie starts from the most ventral portion of the grand average, goes through the microtubule, the transition zone to the microribbon, through the microribbon, then reverses with labeled features: Axis-facing sheet, A; inner sheet, I; margin-facing sheet, M; crossbridge; side rail; bridge; side-arms, SA; paddle; protofilaments are marked (1–13); giardial microtubule inner proteins (gMIP5, gMIP7, gMIP); and giardial microtubule-associated proteins (gMAP1, gMAP2, gMAP3). Scale bar, 5 nm.
(MOV)

Video S6 **Grand average of 4,700 subvolumes by the 16 nm axial repeat at the microtubule/microribbon interface (YZ-plane).** Each step is 0.776 nm. The grand average generated by the software PEET [19], [20] shows the structures associated with ventral disc microtubules. Orientation markers are shown. The movie travels from the axis-facing side, through the lumen of the microtubule, to the margin-facing side. On reverse, the structures are labeled: side-arms, SA; crossbridges, CB; paddle, P; margin-facing sheet, M; inner sheet, I; axis-facing sheet, A; protofilaments are marked (1–13); bridge; giardial microtubule inner proteins (gMIP5, gMIP7, gMIP8); and giardial microtubule-associated proteins (gMIP1, gMIP2, gMIP3). Scale bar, 5 nm.
(MOV)

Video S7 **Isosurface representation of the grand average.** Orientation markers are shown. The isosurface, generated by IMOD [56], is rotated in various directions to point out the structures associated with ventral disc microtubules. The seam is also shown. Protofilaments are numbered (1–13); bridge, B; side rail, SR; axis-facing sheet, A; inner sheet, I; margin-facing sheet, M; crossbridges, CB; side-arms, SA. Scale bar, 5 nm.
(MOV)
