# Correction: A Detailed, Hierarchical Study of *Giardia lamblia*'s Ventral Disc Reveals Novel Microtubule-Associated Protein Complexes

**DOI:** 10.1371/journal.pone.0099456

**Published:** 2014-06-02

**Authors:** The PLOS ONE Staff

There is an error in the ventral disc description of Giardia lamblia. The ventral disc of Giardia lamblia is a right-handed spiral array. Figure 3 legend is incorrect as a result of this error. Please view the correct Figure 3 legend, updated Figure 1, Figure 3, Figure S1, Video S1, Video S2, Video S3, and Video S4, which contains a right-handed spiral array on this page.

**Figure 1:**
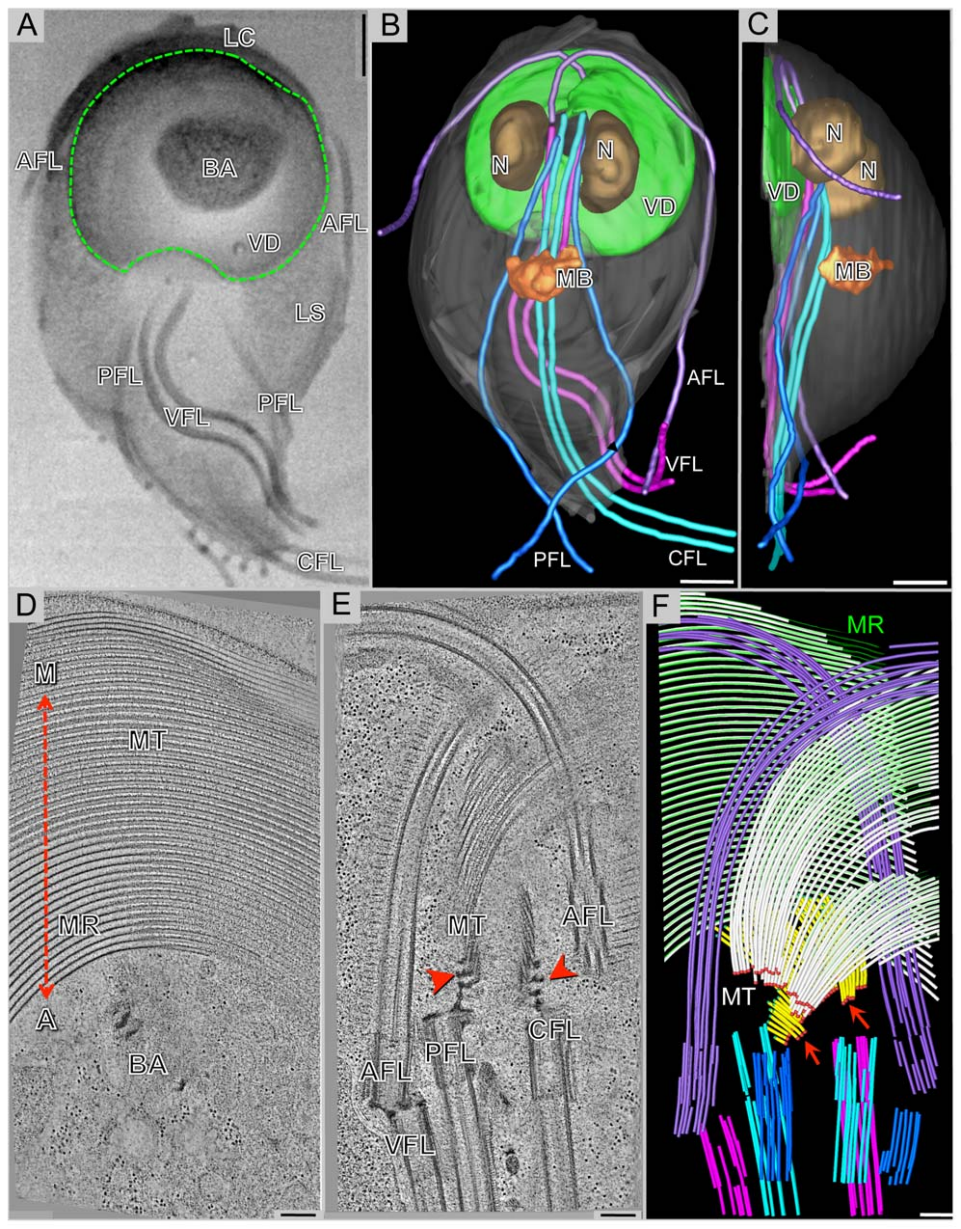
The complex microtubule cytoskeleton of Giardia reconstructed by 3View® and plastic-section tomography. A) Selected SEM slice (back-scattered electron signal) showing eight flagella [anterior flagella (AFL); caudal flagella (CFL); posterior-lateral flagella (PFL); and ventral flagella (VFL)], part of the ventral disc (VD: green outline), the bare area (BA), the lateral shield (LS), and lateral crest (LC). B) 3-D model of a whole-cell reconstruction: ventral disc, nucleus (N), median body (MB), and the four pairs of flagella. C) The side-view of the model shows that the entire microtubule cytoskeleton is located in the ventral part of the cell. D) 5 nm tomographic slice from a montaged, plastic serial section tomogram of a portion of the ventral disc. At the most ventral part of the disc, there are parallel microtubules and microribbons. The relationship of the disc to the helical axis is as indicated: margin-facing (M) or axis-facing (A). The bare area (BA) is also indicated. E) 5 nm tomographic slice showing the arrangement of four basal bodies and how the microtubules (MT) of the ventral disc originate from dense bands (arrows). F) Model from the tomographic reconstruction showing the supernumerary microtubules (yellow) are ventral to the ventral disc microtubules (white). Microtubule ends are classified as either capped (red dots, arrows) or open (green dots). Microribbons are shown in green. One of the anterior flagella (purple) penetrates the overlap zone. Scale bars in A–C  =  2 µm, D–F  =  200 nm.

**Figure 3:**
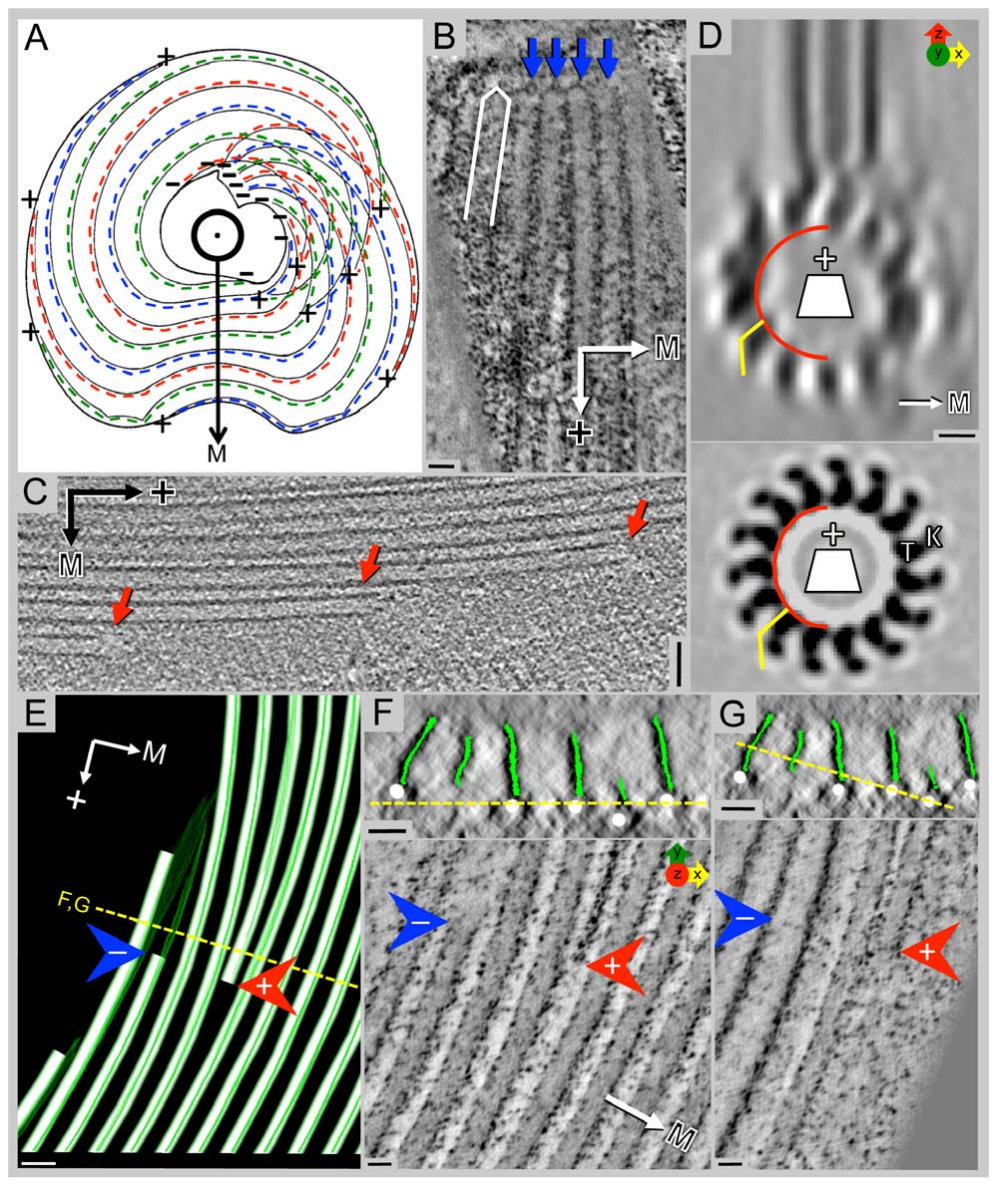
Polarity of ventral disc microtubules is unambiguous with minus-ends originating at dense bands or at the inside edge of the spiral. A) Sketch showing the ventral disc with the dorsal side pointing torward the viewer. Microtubules start with their minus-ends near the overlap zone and spiral downward to the ventral side, thereby forming a right-handed helix. The repetitive units are on the margin-facing side (→M) of the microtubules (dotted colored lines). B) A 10 nm plastic-tomographic slice shows capped microtubule ends (blue arrows) at the dense bands, indicating their minus-ends, while panel C) shows open microtubule ends (red arrows) at the periphery of the disc, typical for plus-ends. D) An end-on view from a helical reconstruction of a bovine microtubule decorated with kinesin-1 motor domains (K) (for example see [61]); when viewed from the minus-end, tubulin (T) shows a right-handed slew while globular microtubule-associated proteins (MAPs) such as kinesin motor domains often bend torward the left (lower panel). The same pattern is visible in the 3-D average of the tomographic reconstruction (upper panel), though with less clarity due to the missing wedge effects. E) A portion of the model of the plastic-section tomogram from Figure 1D showing a plus-end (red arrow) of a microtubule that is ending within the spiral and a minus-end (blue arrow) beginning at the inner edge of the spiral. The microribbons (green) of the inserted microtubules (white) are proximal to the minus-end (blue arrow) of the microtubule. F, G) The upper panels show the plastic-section tomogram in cross-section with the microribbons modeled in green and microtubules in white. The yellow line shows the line of rotation 90° to make the views in the lower panels. The microtubule ending within the spiral (red arrow) is slightly below the neighboring microtubules. The microtubule beginning at the inner edge of the spiral (blue arrow) starts above the neighboring microtubules. Scale bars in B and C  =  50 nm; D  =  5 nm; E  =  50 nm; F and G  =  25 nm. Panel A: adapted with permission from [11].

Additionally, there are some errors in the “Introduction” and “Results” section as a result of the above modification. The correct sentences are listed below:

The second sentence of the third paragraph of the “Introduction” section should read:

“The ventral disc is composed of a right-handed spiral array of parallel microtubules and tightly associated microribbons (Figure 1D) [9–12] that is surrounded by a fibrillar structure called the lateral crest (Figure 1A; LC).”

The fourth sentence of the second paragraph of the sub-heading “Whole cell reconstruction of a Giardia trophozoite illustrates the highly structured 3-D architecture of the microtubule cytoskeleton” of the “Results” section should read :

"The majority of the microtubule-microribbon complexes originates from a series of dense bands at a region near the caudal and posterior-lateral basal bodies (Figure 1E) and form a right-handed spiral (Figure 3A).”

The seventh sentence of the second paragraph of the sub-heading "Whole cell reconstruction of a Giardia trophozoite illustrates the highly structured 3-D architecture of the microtubule cytoskeleton" of the “Results” section should read:

"Occasionally, an array of microtubules called "supernumerary microtubules" [9], which lack associated microribbons and form a short left-handed spiral fragment (Figure 1F)."

## Supporting Information

Figure S1 **Cryo-electron tomography of ventral discs.** A) Isolated cytoskeleton with the ventral disc (VD) and all eight flagella (AFL, CFL, PFL, VFL) present. Areas suitable for cryo-tomography are over the hole in the carbon (box). B) A schematic representation of the ventral disc showing the location of each tomogram used in this study (1–5). Adapted with permission from [11]. C–G) Tomographic slices from each of the tilt-series used to generate the grand average (C, Tomo-1; D, Tomo-2; E, Tomo-3; F, Tomo-4; G, Tomo-5). The left panel is a 25 nm slice through the microtubules and the right panel is a 50 nm slice through the microribbons. Each tomogram is shown with its original orientation with the tilt-axis vertical. In all cases, the 8 nm repeat on the microtubule is obvious (arrow in F, left panel), but the crossbridges between adjacent microribbons are only sometimes seen clearly (arrow in F, right panel). Plus-end and margin directions are indicated. Scale bars in A  =  2 µm, C–G  =  100 nm.
(TIF)

Video S1 **Whole-cell reconstruction of an attached Giardia intestinalis trophozoite using 3View®.** Each slice was obtained using a microtome inside a scanning electron microscope. After each section was removed, a backscatter-signal scanning electron micrograph was recorded [18]. IMOD [56] was used to model important features of the cytoskeleton and attachment sites. Each slice is 70 nm. The majority of organelles are visible with this method: Plasma membrane (grey), median body (orange), nuclei (brown), ventral disc (green), anterior flagella (purple), caudal flagella (cyan), posterior-lateral flagella (blue), and ventral flagella (magenta). Near the ventral portion of the cell, important components of attachment are seen (bare area, lateral crest, lateral shield). Scale bar, 2 µm.
(MOV)

Video S2 **Whole-cell reconstruction model showing relationships between cytoskeletal elements.** The Video starts with the raw data, then transitions into the modeled data created by IMOD [56]. The ventral portion of the cell contains most of the cytoskeletal elements—the ventral disc (VD), median body (MB), and 4 pairs of flagella (anterior flagella, AFL; caudal flagella, CFL; posterior-lateral flagella, PFL; ventral flagella, VFL) as well as the two nuclei (N). The bulk of the cytoskeletal elements are at the ventral portion of the cell–the attachment site to the host microvili. Scale bar, 2 µm.
(MOV)

Video S3 **Tomographic reconstruction of 3 serial-montaged sections of a Giardia trophozoite.** Each slice is 3 nm in the Z-plane. The transition zone between sections looks like a jump. Anterior flagella, AFL (purple); caudal flagella, CFL (cyan); posterior-lateral flagella, PFL (blue); ventral disc microtubules, MT (white); microribbons, MR (green); dense bands, DB; supernumerary microtubules, SMT (yellow). Margin-facing and axis-facing sides are shown for orientation. This volume is about ∼11 µm3 of the entire disc, which has a volume of ∼54 µm3. Scale bar, 500 nm.
(MOV)

Video S4 **Model of tomographic reconstruction of a Giardia trophozoite.** The model was generated using IMOD [56]. The movie starts with the viewer looking torward the ventral surface of the cell. Major cytoskeletal components are present: anterior flagella, AFL (purple); caudal flagella, CFL (cyan); posterior-lateral flagella, PFL (blue); ventral flagella, VFL (magenta); supernumerary microtubules, SMT (yellow); microribbons, MR (green); microtubules, MT (white); open ends are green spheres; closed (capped) ends are red spheres. The overlap zone and dorsal-ventral line are indicated. The movie ends with the viewer looking torward the dorsal surface of the cell. Scale bar, 500 nm.
(MOV)
