# Supplementary figures and images for: A Detailed, Hierarchical Study of Giardia lamblia's Ventral Disc Reveals Novel Microtubule-Associated Protein Complexes
Source: PLoS One. 2012 Sep 11;7(9):e43783. doi: 10.1371/journal.pone.0043783 (PMC3439489; doi:10.1371/journal.pone.0043783)

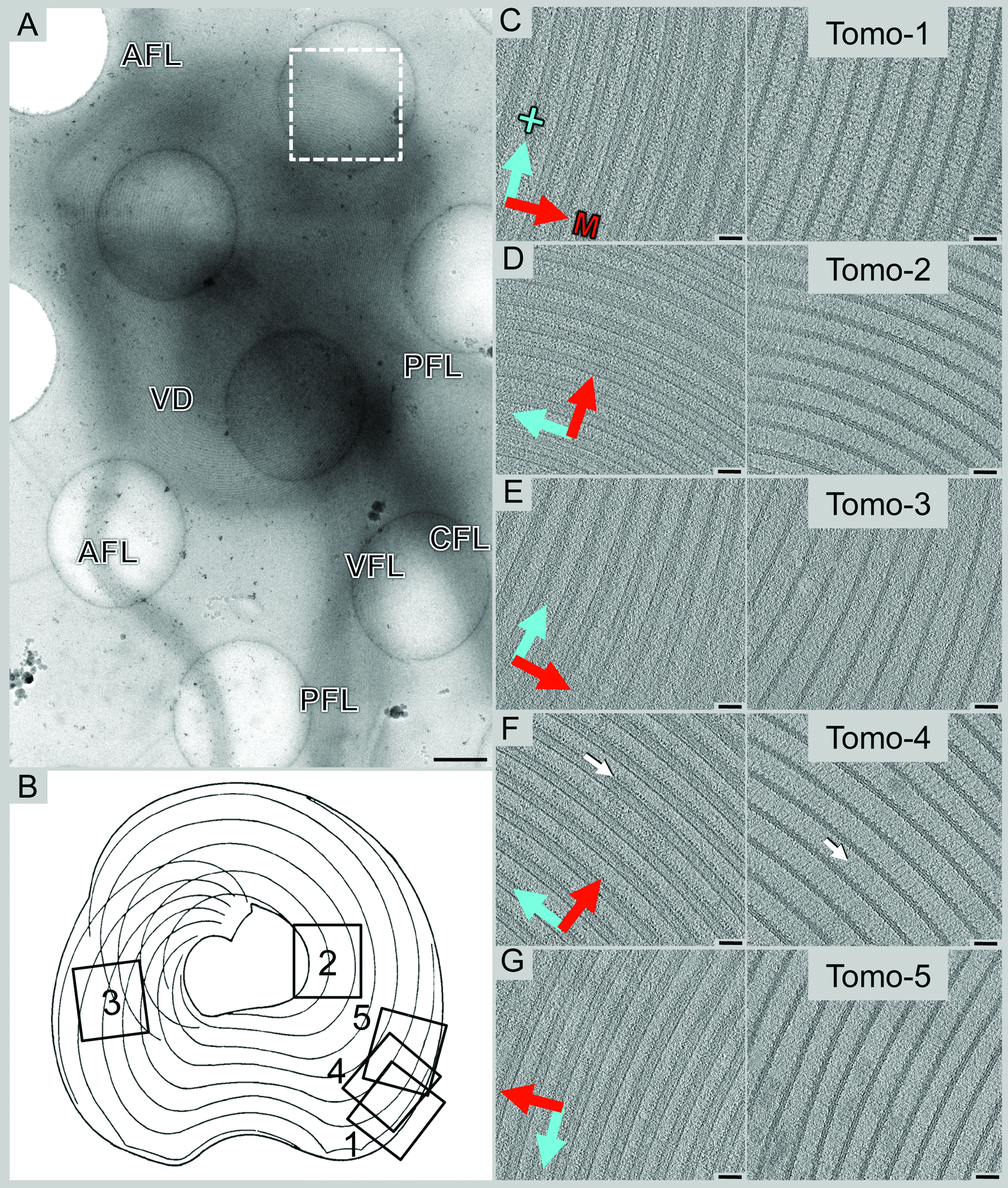

Supplement: Figure S1 — Cryo-electron tomography of ventral discs. A) Isolated cytoskeleton with the ventral disc (VD) and all eight flagella (AFL, CFL, PFL, VFL) present. Areas suitable for cryo-tomography are over the hole in the carbon (box). B) A schematic representation of the ventral disc showing the location of each tomogram used in this study (1–5). Adapted with permission from [11]. C–G) Tomographic slices from each of the tilt-series used to generate the grand average (C, Tomo-1; D, Tomo-2; E, Tomo-3; F, Tomo-4; G, Tomo-5). The left panel is a 25 nm slice through the microtubules and the right panel is a 50 nm slice through the microribbons. Each tomogram is shown with its original orientation with the tilt-axis vertical. In all cases, the 8 nm repeat on the microtubule is obvious (arrow in F, left panel), but the crossbridges between adjacent microribbons are only sometimes seen clearly (arrow in F, right panel). Plus-end and margin directions are indicated. Scale bars in A = 2 µm, C–G = 100 nm. (TIF) [file pone.0043783.s001.tif]

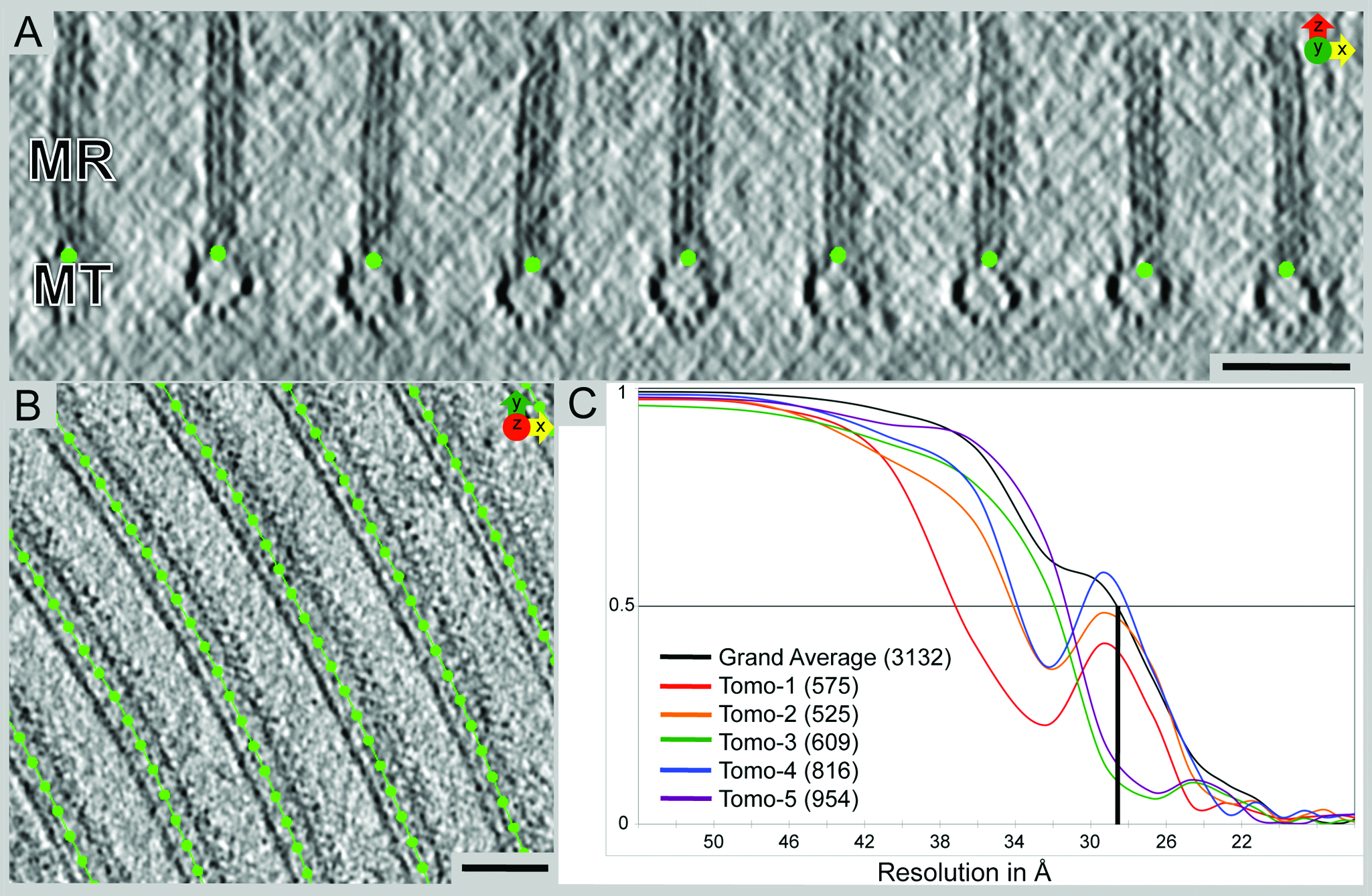

Supplement: Figure S2 — Sub-volume averaging. A, B) Subvolumes were chosen every 16 nm along the axis of the microtubule at the microtubule/microribbon interface. C) Fourier-shell correlation of each individual tomogram average (Tomo-1—Tomo-5) and the grand average. Each number in parentheses shows the number of subvolumes used to calculate the Fourier-shell correlation. The grand average has a resolution of ∼28 Å. (TIF) [file pone.0043783.s002.tif]

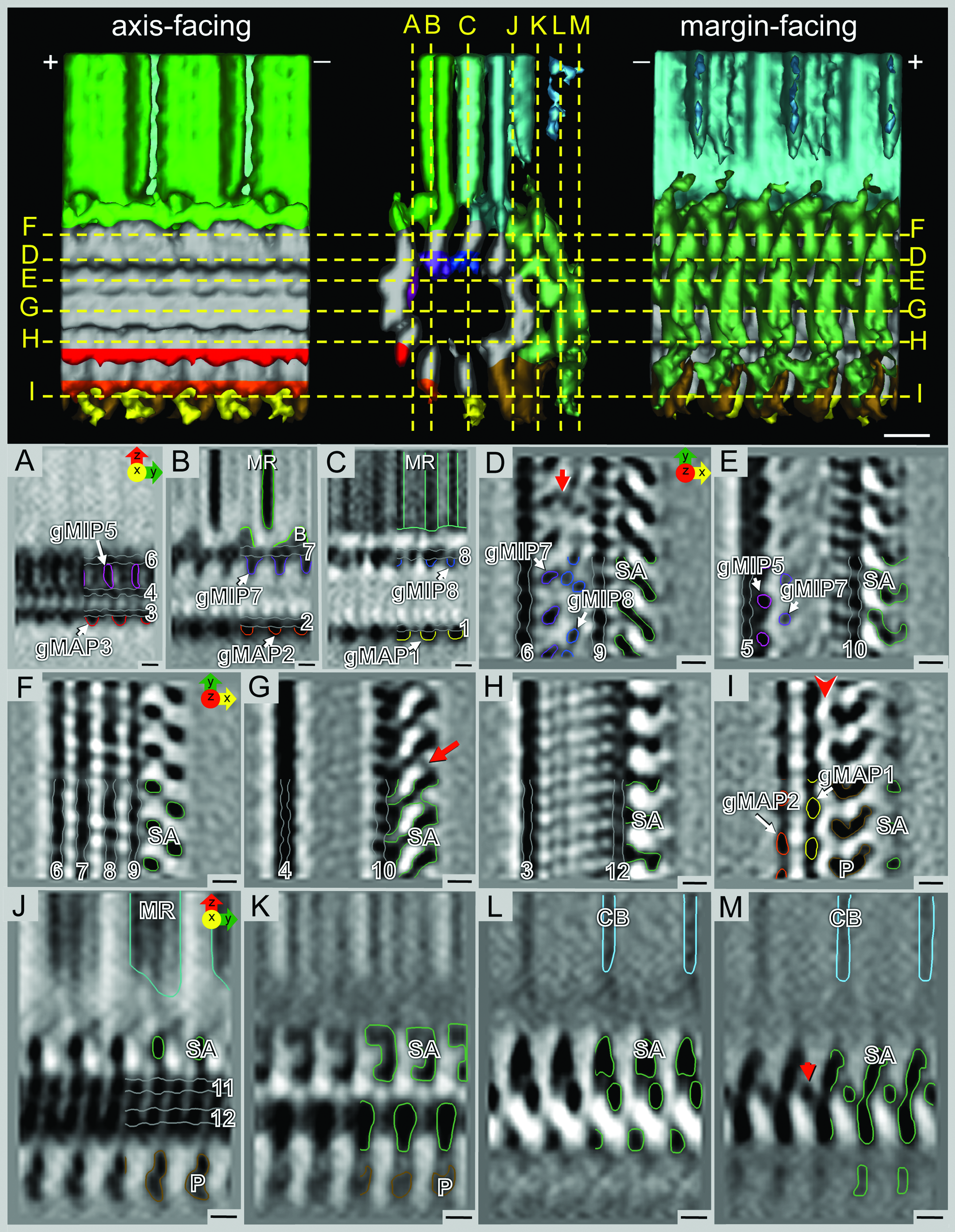

Supplement: Figure S3 — Giardial Microtubule Inner Proteins (gMIPs) and Giardial Microtubule-Associated Proteins (gMAPs) are key features of ventral disc microtubules. A) gMIP5 (magenta) has an 8 nm repeat and is found on the inside surface of protofilament 5 (PF5), spanning the distance between PF4 and PF6. gMAP3 (red) occurs every 8 nm on PF3. B) gMIP7 (purple) is attached to PF7 every 8 nm. Part of the bridge (B; green) is attached to the outside surface of PF7. gMAP2 (orange) occurs every 8 nm and is attached to PF2. C) gMIP8 (blue) is attached to PF8 and has a 16 nm repeat of three globular domains (2 are shown in this slice). gMAP1 (yellow) is found every 8 nm and is attached to PF1. D) In the XY orientation, it is clear that gMIP8 has a 16 nm repeat consisting of three different densities. There is a possible lateral connection between gMIP7 and gMIP8 (arrowhead). E) Two of the three gMIPs are shown. gMIP5 (magenta) has an 8 nm repeat and is found on the inside surface of PF5. gMIP7 (purple) has an 8 nm repeat and is found on PF7. F) Side-arms follow an 8 nm repeat. Near the top of the side-arm, a portion is attached to PF9. G) The side-arm is attached to PF10 and has lateral connections between neighboring side-arms (arrow). H) Side-arms are attached to PF12. I) A portion of the side-arm has been differentiated as the paddle (brown). The seam of the microtubule is located between gMAP1 and the paddle (arrowhead). J–M) YZ-slices showing how the side-arms (SA) follow the helix of the microtubule. (J) Side-arms attach to the microtubule at PF10 and (K) PF12. The paddle attaches to PF13. (L) The beginning of the cross-bridges where they are attached to the marginal-facing sheet. (M) The same linker as in G between adjacent side-arms (arrow). Scale bars, 5 nm. (TIF) [file pone.0043783.s003.tif]
