# Supplementary figures and images for: Correction: A Detailed, Hierarchical Study of Giardia lamblia's Ventral Disc Reveals Novel Microtubule-Associated Protein Complexes
Source: PLoS One. 2014 Jun 2;9(6):e99456. doi: 10.1371/journal.pone.0099456 (PMC4041866; doi:10.1371/journal.pone.0099456)

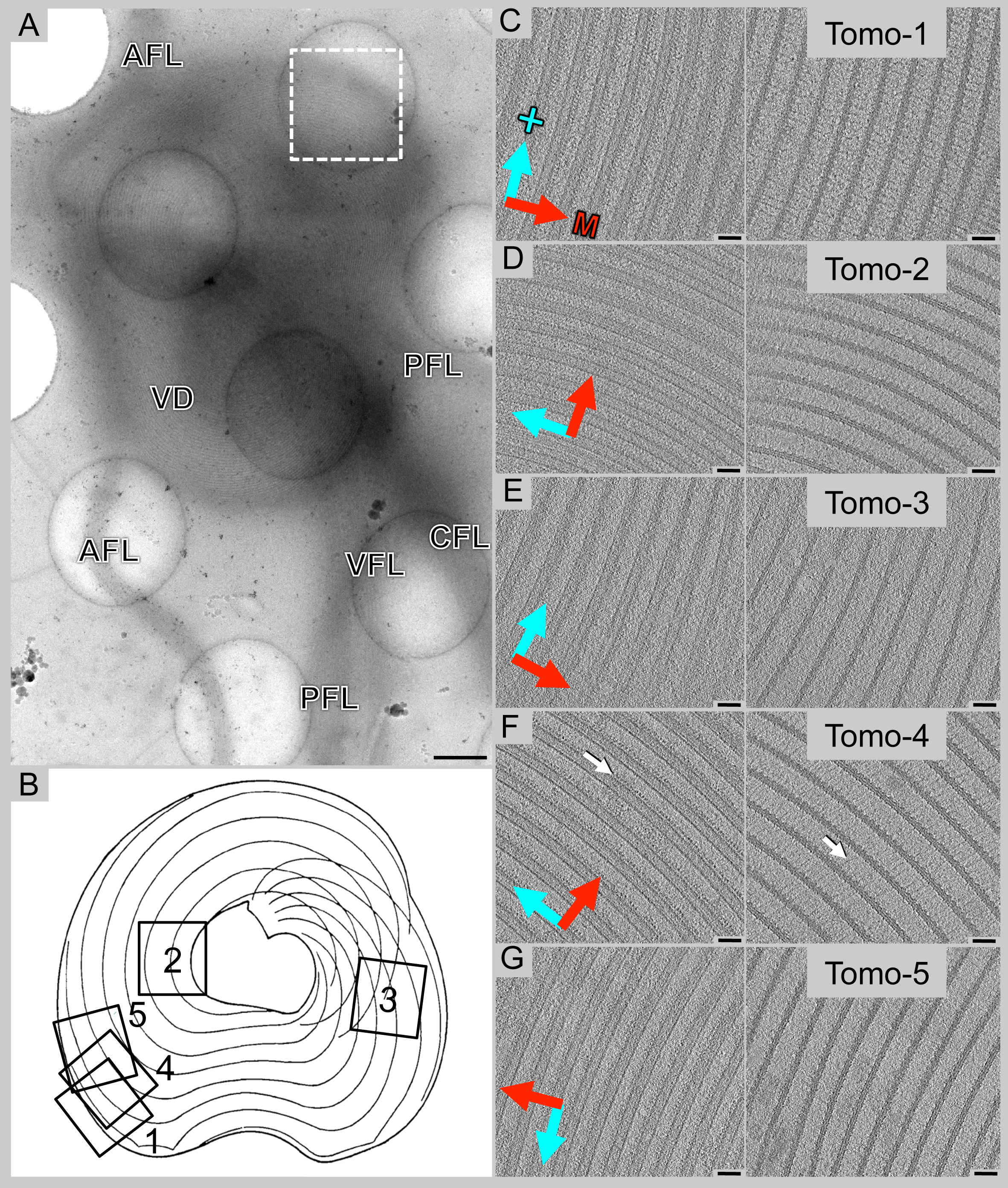

Supplement: Figure S1 — Cryo-electron tomography of ventral discs. A) Isolated cytoskeleton with the ventral disc (VD) and all eight flagella (AFL, CFL, PFL, VFL) present. Areas suitable for cryo-tomography are over the hole in the carbon (box). B) A schematic representation of the ventral disc showing the location of each tomogram used in this study (1–5). Adapted with permission from [11]. C–G) Tomographic slices from each of the tilt-series used to generate the grand average (C, Tomo-1; D, Tomo-2; E, Tomo-3; F, Tomo-4; G, Tomo-5). The left panel is a 25 nm slice through the microtubules and the right panel is a 50 nm slice through the microribbons. Each tomogram is shown with its original orientation with the tilt-axis vertical. In all cases, the 8 nm repeat on the microtubule is obvious (arrow in F, left panel), but the crossbridges between adjacent microribbons are only sometimes seen clearly (arrow in F, right panel). Plus-end and margin directions are indicated. Scale bars in A = 2 µm, C–G = 100 nm. (TIF) [file pone.0099456.s001.tif]
